# NMR-Based Metabolic Profiling of Field-Grown Leaves from Sugar Beet Plants Harbouring Different Levels of Resistance to *Cercospora* Leaf Spot Disease

**DOI:** 10.3390/metabo7010004

**Published:** 2017-01-26

**Authors:** Yasuyo Sekiyama, Kazuyuki Okazaki, Jun Kikuchi, Seishi Ikeda

**Affiliations:** 1 Food Research Institute, National Agriculture and Food Research Organization (NARO), Tsukuba 305-8642, Japan; 2 RIKEN Center for Sustainable Resource Science, 1-7-22 Suehiro-cho, Yokohama 235-0045, Japan; jun.kikuchi@affrc.go.jp; 3 Hokkaido Agricultural Research Center, NARO 9-4 Shinsei-minami, Memuro 082-0081, Japan; okakazu@affrc.go.jp (K.O.); sikeda67@affrc.go.jp (S.I.); 4 Graduate School of Medical Life Sciences, Yokohama City University, Yokohama 230-0045, Japan; 5 Graduate School of Bioagricultural Sciences, Nagoya University, Nagoya 464-8601, Japan

**Keywords:** NMR, metabolomics, sugar beet (*Beta vulgaris* L.), *Cercospora* leaf spot disease

## Abstract

*Cercospora* leaf spot (CLS) is one of the most serious leaf diseases for sugar beet (*Beta vulgaris* L.) worldwide. The breeding of sugar beet cultivars with both high CLS resistance and high yield is a major challenge for breeders. In this study, we report the nuclear magnetic resonance (NMR)-based metabolic profiling of field-grown leaves for a subset of sugar beet genotypes harbouring different levels of CLS resistance. Leaves were collected from 12 sugar beet genotypes at four time points: seedling, early growth, root enlargement, and disease development stages. ^1^H-NMR spectra of foliar metabolites soluble in a deuterium-oxide (D_2_O)-based buffer were acquired and subjected to multivariate analyses. A principal component analysis (PCA) of the NMR data from the sugar beet leaves shows clear differences among the growth stages. At the later time points, the sugar and glycine betaine contents were increased, whereas the choline content was decreased. The relationship between the foliar metabolite profiles and resistance level to CLS was examined by combining partial least squares projection to latent structure (PLS) or orthogonal PLS (OPLS) analysis and univariate analyses. It was difficult to build a robust model for predicting precisely the disease severity indices (DSIs) of each genotype; however, GABA and Gln differentiated susceptible genotypes (genotypes with weak resistance) from resistant genotypes (genotypes with resistance greater than a moderate level) before inoculation tests. The results suggested that breeders might exclude susceptible genotypes from breeding programs based on foliar metabolites profiled without inoculation tests, which require an enormous amount of time and effort.

## 1. Introduction

*Cercospora* leaf spot (CLS), which is caused by the fungus *Cercospora beticola* Sacc., is one of the most serious leaf diseases for sugar beet (*Beta vulgaris* L.) worldwide [1,2]. As the disease progresses, infected leaves exhibit numerous leaf spot lesions, which lead to complete leaf collapse. The loss of mature leaves and growth of new leaves considerably reduce both the root yield and recoverable sucrose. The introduction of CLS-resistant cultivars in a breeding program is an important strategy for controlling plant disease. Resistance to CLS is characterised by an inherited quantitative trait and is rate-limiting with respect to disease development [3,4]. In addition, the quantitative resistance is characterised by a strong defence response at a later stage of the infection cycle (i.e., the formation of necrotic lesions) [5]. The quantitative trait loci (QTL) of CLS resistance have been mapped in a number of previous studies to establish a marker-assisted selection system [2,6,7]. Genes in pathogen recognition, cell signalling, and defence-related proteins (e.g., those with chitinase or glucanase activity) are all involved in quantitative resistance against *C. beticola* [5]. However, despite these efforts, the resistance mechanism remains unclear. In the absence of pathogen, existing cultivars having higher resistance generally possess a lower root yield potential. Therefore, the breeding of new cultivars that possess both high CLS resistance and high yield potential remains a major challenge for breeders. However, no efficient screening of sugar beet for CLS resistance has been proposed, mainly because, under field conditions, diverse environmental factors often strongly affect the expression of quantitative resistance. For example, conidia are spread by wind, water (irrigation and rain), and insects during warm and humid weather, which affects the inoculum density of the pathogen [8]. It is also conceivable that the physiological state of plants, such as leaf age and a characteristic of stomatal opening and closing, considerably influences disease severity [9].

A better understanding of the plant metabolism response to environmental stresses, including pathogen infections, can help us understand plant physiology, which is crucial for the future applications of plant metabolomics in plant breeding and disease control. However, metabolomic studies of sugar beet are still rare [10]. Metabolite profiling by gas chromatography mass spectrometry (GC-MS) has been used for sugar beet root and characterises the response to iron deficiency of the carbohydrate metabolism and tricarboxylic-acid cycle [11]. Wound-induced changes in the primary carbon metabolism in sugar beet root were also determined by Lafta et al. [12]. Furthermore, Webb et al. reported ultra-performance liquid chromatography (UPLC)-MS and GC-MS-based metabolomics to characterise the defence response of sugar beet leaves and roots to *Rhizoctonia solani* AG 2-2 IIIB [13]. Antioxidants, such as polyphenols, in fresh sugar beet root and fermented beetroot juice were fingerprinted by UPLC-MS and organic and conventional production was compared [14]. Comprehensive measurements of metabolites can also help shed light on the underlying mechanism of CLS resistance in relation to various environmental factors in the field. This approach may also facilitate an assessment of CLS resistance by developing metabolic markers, potentially, in a manner complementary to DNA-marker-assisted breeding. Indeed, metabolite profiling has recently become an increasingly popular means to assess the physiological status of crops under field conditions [15]. 

Among the analytical techniques available for metabolomics, NMR analysis offers a series of advantages for obtaining an overview of all detectable small molecules and for obtaining robust, reproducible data. We recently reported non-targeted NMR-based metabolic profiling of field-grown leaves from potato plants that harboured different levels of resistance against late blight disease [16]. This work allowed us to find the resistance-related metabolite, l-malic acid, and to develop an enzymatic assay for screening of resistant cultivars as an example of a simple and cost-effective method. In the present study, we apply NMR-based metabolic profiling to field-grown leaves from sugar beet plants harbouring different levels of CLS resistance.

## 2. Results

### 2.1. Annotation of Aqueous Metabolites in Sugar Beet Leaves

Table 1 lists 12 genotypes used in the present study and the disease severity indices (DSIs) recorded in 2015. Stout, Monohikari, and Lemiel are the standard cultivars with clearly different and stable CLS resistance levels, and have been used for resistance tests at Hokkaido Agricultural Research Centre (HARC) over ten years as references of strongly-, moderately-, and weakly-resistant (susceptible) cultivars. The daily climate data are shown in Supplementary Figure S1. Initially, we annotated the ^1^H-NMR signals of sugar beet leaves. Very few NMR spectra have been reported for metabolite mixtures in sugar beet [17,18]. To the best of our knowledge, for the metabolome in sugar beet leaves, only one research group (Institut National de la Recherche Agronomique, France) has reported NMR spectra with detailed metabolite information [19]. A D_2_O-based potassium phosphate buffer (KPi) was used to extract aqueous metabolite mixtures at 90 °C from lyophilised leaf powder. In this study, we focused on the water-soluble and stable metabolites as the first step of field metabolomics, so that breeders can easily manipulate the metabolite markers in the future. Supplementary Figure S2 shows ^1^H-NMR spectra of the susceptible (Ezomaru) and resistant (NK-310mm-O) sugar beet leaves. Thirty metabolites in sugar beet leaves were annotated as follows: sucrose (Suc), glucose (Glc), fructose (Fru), acetate, malate (MA), succinate (SA), citrate (CA), fumarate, formate, isoleucine (Ile), leucine (Leu), valine (Val), threonine (Thr), alanine (Ala), proline (Pro), glutamate (Glu), glutamine (Gln), aspartate (Asp), aspargine (Asn), 4-aminobutyrate (GABA), tyrosine (Tyr), tryptophan (Trp), phenylalanine (Phe), methanol (MeOH), ethanolamine, choline, glycine betaine (betaine), adenine, adenosine triphosphate (ATP), and trigonelline (Trg).

### 2.2. Effects of Growth Stage on Metabolite Profile in Sugar Beet Leaves

**Figure 1:**
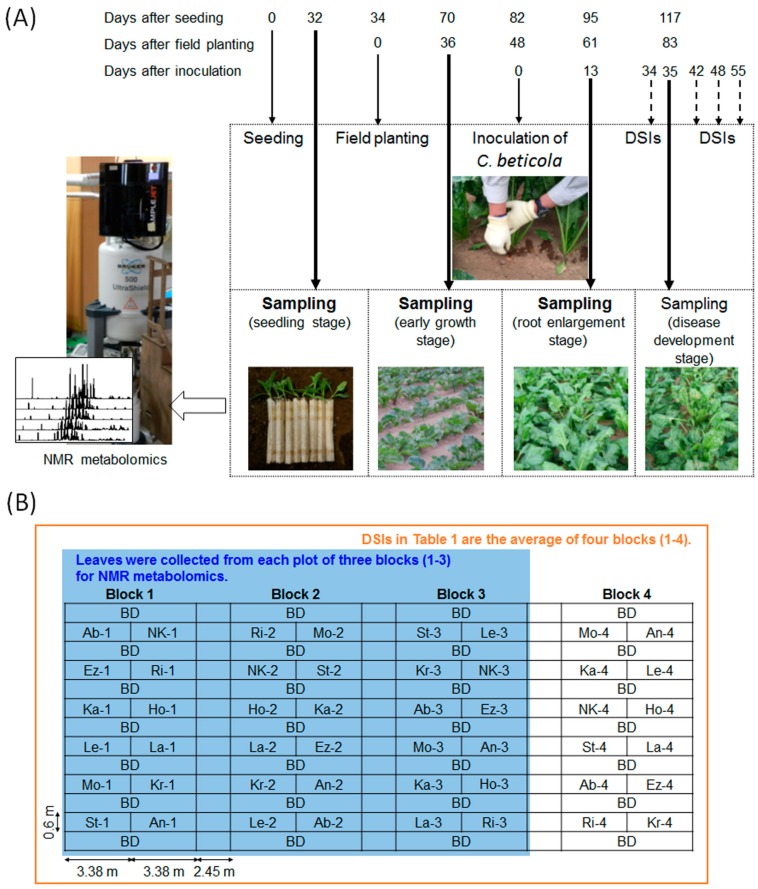
(**A**) Sampling timetable during field testing of *Cercospora* leaf spot (CLS) resistance. The thin, bold, and dashed arrows indicate the schedules for cultivation, sampling time points, and recording disease severity indices (DSIs), respectively; (**B**) Block design for the field test. The individual plot size for each genotype was 2.03 m^2^ (3.38 m × 0.6 m), and the plant density was 15 plants per plot. Each plot is described by genotype abbreviation and block number. For NMR metabolomics, leaves were collected from one plant in each plot of three blocks as samples 1–3. A DSI was given to each plot of four blocks and a DSI for a sugar beet genotype in Table 1 was calculated by averaging DSIs across four blocks. BD: uninoculated border plots of the highly resistant cultivar Rivolta (Ri).

**Figure 2:**
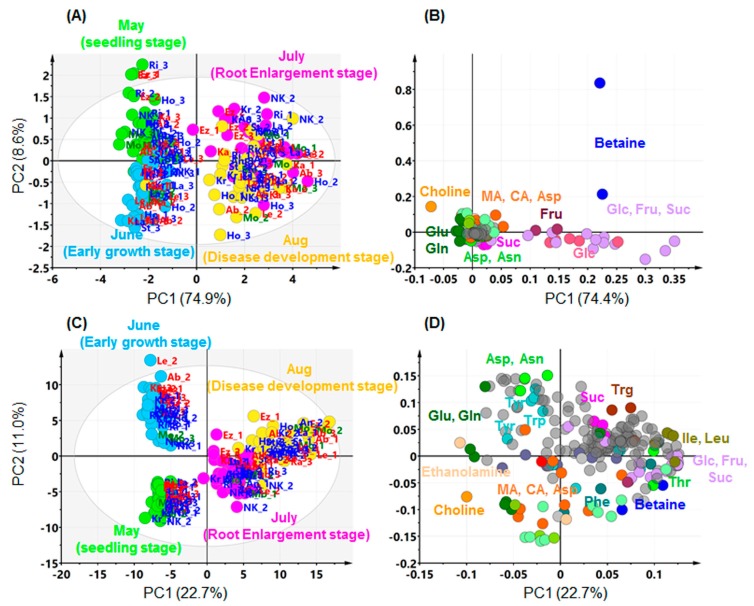
Principal component analysis (PCA) score (**A**,**C**) and loading (**B**,**D**) plots of the NMR data of sugar beet leaves. Pareto (**A**,**B**) or unit variance (**C**,**D**) scaling was applied to the NMR buckets. The abbreviations of genotypes harbouring weak (red), moderate (green), and strong (blue) resistance levels are defined in Table 1. Grey circles in the loading plots indicate unannotated NMR buckets.

The leaves of 12 sugar beet genotypes harbouring different resistance levels to CLS were collected at four different sampling time points: the seedling stage (just prior to planting in an experimental field), the early growth stage (approximately one month after transplanting), the root-enlargement stage (approximately two months after transplanting and two weeks after inoculation with *C*. *beticola*), and the disease-development stage (approximately one month after inoculation with *C*. *beticola*). Figure 1 shows the sampling timetable and block design during the field test. The entire NMR dataset from the four growth stages was subjected to an exploratory unsupervised multivariate method—principal component analysis (PCA; Figure 2). NMR variables consisting of fixed-size (0.04 ppm) integral bin areas (buckets) were pre-processed by either Pareto scaling or unit variance scaling (autoscaling) prior to analysis. Pareto scaling gives a greater weight to large variables, while unit variance scaling gives each variable equal weight. The Pareto-scaled data stays closer to the original value and, therefore, the interpretation of loadings from PCA is relatively straightforward. Although the influence of noise, caused by experimental or measurement errors, is larger in unit variance scaling, this scaling method can eliminate bias towards large signals and detect small, but important, signals. The number of significant components and explained variance (cumulative *R*^2^*X*) were 12 and 0.98 when Pareto scaling was applied, while these were 11 and 0.63, respectively, when unit variance scaling was applied. In both scaling methods, PC1 was responsive to the sampling time points and differentiated between the earlier and later halves of the growth stage. The loading plots indicated that this separation was primarily explained by the abundance of major metabolites, such as sugars, betaine, and choline. By the later growth stage, the dominant sugars, Suc, Glc, Fru, and betaine, have accumulated in sugar beet leaves, whereas choline has decreased. Changes in minor metabolites were also detected by unit variance scaling. At the later growth stage, Glu, Gln, and ethanolamine were decreased, while Leu and Ile were increased. PC2 and PC3 also showed a trend to separate growth stages, i.e., PC2 was somewhat related to the difference between the seedling stage and the early growth stage, while PC3 was related to the difference between the root enlargement stage and the disease-development stage. The score and loading plots of the PC3 versus the PC4 plane are shown in Supplementary Figure S3. No clear trends relating to CLS resistance appeared in any other components. The results suggested that the metabolic markers of CLS resistance in sugar beet leaves, if any, might vary according to the growth stage or environmental factors, such as elevated temperature and humidity, at each sampling point. 

### 2.3. Relation between Leaf Metabolites and Disease Severity

For the next step, we examined the relationship between foliar metabolite profiles and levels of resistance to CLS. Based on the results of PCA, partial least squares projection to latent structure (PLS) and orthogonal PLS (OPLS) analyses were applied separately to each growth stage. The DSIs recorded at four time points (see Table 1) were used as *Y* variables. The number of significant components, goodness-of-fit parameter (*R*^2^), and the fraction correctly predicted in the model (*Q*^2^) in a 1/7th cross-validation are provided in Supplementary Tables S1 (PLS) and S2 (OPLS). These parameters are most frequently used to assess the model performance in multivariate analysis [21,22,23]. The result suggested that the early growth stage in June could be a better sampling point to examine the metabolite profiles relating to CLS resistance. However, the largest *Q*^2^ value of the PLS models was 0.5, which is widely admitted as a significance threshold for *Q*^2^ [21], although actually it is difficult to determine the general limit of *Q*^2^. For internal validation of the models, a permutation test (*n* = 500) was performed. The *Y* variables were randomly re-ordered 500 times, while the *X*-matrix was kept constant. Each time a new model was fitted and *R*^2^ and *Q*^2^ were plotted against the correlation coefficient between the original *Y* vector and the permuted *Y* vector. For the model to be valid, all of the *R*^2^ and *Q*^2^ values on the permuted dataset must be lower than those values on the actual dataset [24]. The intercept values of regression lines for *R*^2^ and *Q*^2^ were shown in Tables S1 and S2. The numbers of permuted *R*^2^ and *Q*^2^ values exceeding original values for PLS and OPLS models are shown in Tables S3 and S4. The result indicate that the models have a significant degree of overfitting, especially at the root-enlargement stage. Therefore, it will be difficult to predict precisely the DSIs of new genotypes according to the models obtained in this study. The variable importance in the projection (VIP) scores obtained from the unit variance-scaled data and the important NMR buckets (rank 1–20) are listed in Tables S5–S8 (PLS) and Tables S9–S12 (OPLS). We also examined the correlation between the original intensity of each NMR bucket (normalised to the intensity of the internal standard, DSS) and the DSI values. Pearson’s correlation coefficient and the buckets with |*r*| > 0.5 are listed in Table S13. No significant linear correlation was observed between DSIs and NMR spectra. Therefore, we tried to find candidate metabolite signatures to distinguish two genotype groups defined as susceptible (four genotypes with weak resistance) and resistant (one genotype with moderate resistance and seven genotypes with strong resistance), since this may be useful for breeders if they can exclude weak lines during the breeding program. Welch’s *t*-test [25] was performed on each NMR bucket (original intensity as used for correlation analysis) between the susceptible and resistant groups. The NMR buckets with the highest VIP values (rank 1–20), Pearson’s correlation coefficient |*r*| > 0.5 and Welch’s *t*-test *p*-value less than 0.05 were picked as candidate marker buckets and and used for further examination. The important buckets selected by PLS (Table 2) and OPLS (Table S13) seems to contain substantially the same metabolites. The unannotated buckets of 5.04–5.00 and 5.72–5.68 ppm did not allow further structural analysis by two dimensional (2D) NMR due to low concentrations. A candidate marker bucket at the seedling stage was the unannotated region at 3.96–3.92 ppm. Two-dimensional ^1^H-^13^C heteronuclear quantum coherence (HSQC) spectra suggested that the bucket contains at least three alcohol signals (likely to be sugars): δ_H_ = 3.92; δ_C_ = 71.4, δ_H_ = 3.93; δ_C_ = 73.0, δ_H_ = 3.95; δ_C_ = 79.3. At the early growth stage, GABA, Asn, Glu, MA, Gln, Pro, Val, acetate, and unannotated minor metabolites in the bucket of 5.04–5.00 ppm might be responsive to CLS resistance. The candidate markers at the root enlargement stage were Gln and unannotated metabolites in the three buckets of 5.04–5.00, 5.72–5.68, and 8.00–7.96 ppm. The bucket of 8.00–7.96 ppm consists of part of a doublet signal (*J* = 8.1 Hz) at 7.98 ppm, coupled with a doublet signal (*J* = 8.1 Hz) at 7.04 ppm, and a small singlet signal at 8.00 ppm. ^1^H-^13^C HSQC, ^1^H-^1^H double-quantum-filtered correlation spectroscopy (DQF-COSY) and total correlation spectroscopy (TOCSY) spectra indicated that three analogous aromatic compounds, which may belong to para-substituted phenylpropanoid, are included in sugar beet leaves, and that two of these are more abundant in resistant cultivars (Figure S4). At the disease development stage, Suc, Fru, and the buckets with 3.96–3.92 and 8.00–7.96 ppm might differentiate between the susceptible and resistant groups. 

### 2.4. Comparing Annotated Candidate Metabolites

**Figure 3:**
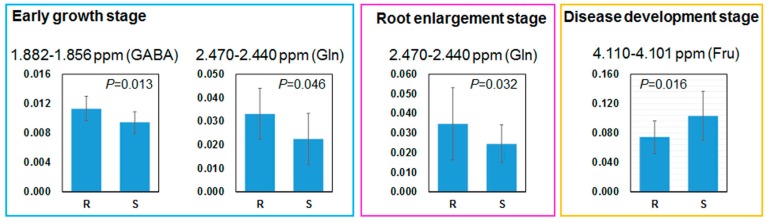
Relative intensity of the isolated NMR regions annotated to 4-aminobutyrate (GABA) (1.882–1.856 ppm) and glutamine (Gln) (2.470–2.440 ppm) at the early growth stage, Gln at the root enlargement stage, and Fru (4.316–4.290 ppm) at the disease development stage. S: susceptible group; and R: resistant group.

The candidate NMR buckets prepared by fixed-size binning, as described above, include overlapped signals from multiple metabolites. Therefore, we also tried to compare partially isolated signal regions of the eight annotated metabolites. The signal regions were selected to include single annotated metabolites as far as possible: GABA (1.882–1.856 ppm, a part of the quintet signal of C-3 methylene), Asn (2.844–2.840 ppm, a part of the double doublet signal of C-3 methylene), Glu (2.058–2.029 ppm, a part of the double doublet signal of C-3 methylene; the Pro signals at C-2 and C-3 are slightly overlapped), MA (4.316–4.290 ppm, a part of the double doublet signal of C-2 methine), Gln (2.470–2.440, a part of the multiplet signal of C-4 methylene), Pro (1.992–1.980 ppm, a part of the multiplet signal of C-4 methylene), Val (1.040–1.017 ppm, a part of the doublet signal of C-4 methyl), Suc (5.422–5.382 ppm, the doublet signal of anomeric proton of the Glc moiety), and Fru (4.110–4.101 ppm, a part of the multiplet signal of C-3 and C-4 methine) were manually integrated and normalised to DSS. The singlet signal of acetate at 1.90 ppm was completely overlapped with the signal of GABA and could not be isolated. The intensities of eight signal regions were compared by Welch’s *t*-test between susceptible and resistant groups. Significant differences were observed in GABA and Gln at the early growth stage, Gln at the root enlargement stage, and Fru at the disease development stage (Figure 3). In the susceptible group, GABA has a tendency to be lower at the early growth stage, while Fru is higher at the disease development stage. Gln was lower in the susceptible group at both the early growth and root enlargement stages. These isolated regions were also compared among 12 genotypes using Tukey’s multiple comparison test (Figure S5). As expected by the PLS, OPLS and correlation analyses described above, it was difficult to predict the order of resistance levels for each genotype by these metabolites. However, we believe that it may be useful for breeders if they can exclude weak lines without the inoculation test, which requires an enormous amount of time and effort.

## 3. Discussion

In the present study, we applied NMR-based metabolic profiling of field-grown leaves to a subset of sugar beet (*Beta vulgaris* L.) genotypes harbouring different levels of CLS resistance. The dominant metabolites, Suc, Glc, Fru, and betaine, accumulated in leaves at a later growth stage, while choline, which is the biosynthetic precursor of betaine, was reduced in content. Concerning minor metabolites, Glu, Gln, and ethanolamine were decreased, while Leu and Ile were increased at the later growth stage. It was conceivable that the sugar beet leaves accumulated Suc towards the development and maturation of roots. It was reported that mature leaves of drought-stressed sugar beet accumulate sucrose [26]. Similarly, sucrose accumulation in our study could be attributable to drought stress; however, this could not be confirmed by the climate data for 2015 (Figure S1). Glycine betaine is also known to accumulate in response to abiotic stress in many crops, including sugar beet [27]. The betaine accumulation at a later growth stage might also be attributable to abiotic stress, such as elevated temperature. Mäck et al. reported that the concentration of Suc, Glc, Fru, and Gln in different organs, i.e., beet, crown, and young and mature leaf petioles and blades, changes depending on the yearly variation in precipitation [28]. Therefore, we need to perform year-to-year comparisons of the field test.

We also examined the relationship between foliar metabolite profiles and levels of resistance to CLS by combining PLS or OPLS and univariate analyses. Unfortunately, no clear linear correlation could be found between foliar metabolites and DSIs during a four-month field trial. Therefore, it is difficult to predict precisely the DSIs of new genotypes according to the metabolic changes found in this study. However, it was suggested that GABA, Gln, and Fru roughly differentiate susceptible genotypes (genotypes having weak resistance) from resistant genotypes (genotypes having resistance higher than moderate level). In our sugar beet field trial, GABA and Gln were lower in quantity in the leaves of the susceptible genotypes before pathogen attack. These results suggested that breeders could exclude at least weak genotypes from breeding programs based on sugar beet foliar metabolites before the inoculation test, which requires an enormous amount of time and effort. In this study, we examined polar and stable primary metabolites that are soluble in water as the first step of a field metabolomics trial. Such metabolites seem to be easily accessible for breeders. In future challenges, we need to determine the structures of the unannotated aromatic metabolites, which may belong to para-substituted phenylpropanoid, at 8.00–7.96 ppm. Furthermore, we should explore semi-polar metabolites, nonpolar metabolites, or bioactive secondary metabolites, which may also be useful markers to CLS resistance. Field conditions, which are highly heterogeneous and inconsistent over years, affect plant metabolism and disease severity. Therefore, repetition of experiments over years is required using a wide range of sugar beet cultivars. A further and long-term field trial has now started.

## 4. Materials and Methods

### 4.1. Preparation of NMR Samples

#### 4.1.1. Plant Materials and Sampling

Sugar beet plants were grown in 2015 according to the conventional field management strategy described by Taguchi et al. [6] for an experimental field at the Hokkaido Agricultural Research Centre (HARC). Table 1 lists eleven cultivars and one breeding line (NK-310mm-O) used in the present study with their sources and DSIs for CLS observed in 2015. We used Tukey’s multiple-comparison test with R software (ver. 3.1.2; R Core Development Team, Vienna, Austria), with a 0.05 level of probability as the criterion determining significant DSI differences between sugar beet genotypes. Sugar beet seeds were sown in paper pots (19 mm diameter and 13 cm high, Nippon Beet Sugar Mfg. Co., Ltd., Obihiro, Japan) on 9 April 2015, and the seedlings were grown in a greenhouse. On 13 May 2015, the seedlings were transplanted to an experimental field at HARC (Memuro, Hokkaido, Japan; 42°89.2′ N/143°0.7.7′ E, 92 m a.s.l.). The field was dressed with S014 (150, 315 and 210 kg/ha for N, P_2_O_5_, and K_2_O, respectively; Hokuren Fertilizer Co., Sapporo, Japan) as a basal fertilizer. The daily climate data (mean air temperature, mean relative humidity, and mean rainfall) are shown in Figure S1. The field experiment was arranged in a randomised block design with four replications (Figure 1B). The individual plot size for each genotype was 2.03 m^2^ (3.38 m × 0.6 m) and the plant density was 15 plants per plot. Inoculation with *C*. *beticola* and resistance evaluation was also conducted following the procedure established in a previous study [6]. Briefly, dried powder of leaves infected with *C*. *beticola* collected from the HARC fields in the previous year was applied at the foot of each plant on 30 July 2015 (48 days after transplanting). Symptoms of CLS were rated based on a visual index ranging from zero (no symptoms) to five (main leaves fully destroyed). A DSI was determined for each plot and averaged across four block replicates for a sugar beet genotype. The DSIs were recorded at four time points: 3, 11, 17, and 24 August (i.e., 34, 42, 48, and 55 days after inoculation, respectively). The leaves for NMR analyses were collected from 12 sugar beet genotypes at four time points: (i) at the seedling stage, just prior to planting in the experimental field (11 May; 32 days after seeding); (ii) at the early growth stage (18 June; 36 days after transplanting); (iii) at the root-enlargement stage (13 July; 61 and 13 days after transplanting and inoculation with *C. beticola*, respectively); and (iv) at the beginning of August during the disease development stage (4 August, 83 and 35 days after transplanting and inoculation, respectively). At the seedling stage, all leaves were collected from three plants to form a composite sample (this approach was taken due to the small amount of samples). Three composite samples were prepared from nine plants grown in a greenhouse as triplicate samples for metabolomics. After transplanting into the experimental field, three fully-expanded leaves were collected from an individual plant in a plot as a sample and three samples were obtained from each of three blocks (1–3) as triplicates. In this way, a total of 144 samples (12 genotypes × 3 composite seedling samples or plant replicates × 4 sampling time points) were collected. These leaves were freeze-dried in a lyophiliser (FD-20BU/SK01; Nihon Techno Service Co., Ltd., Ibaraki, Japan) and then ground into a fine powder by using a Shake Master (Bio Medical Science, Inc., Tokyo, Japan).

#### 4.1.2. Metabolite Extraction

To profile polar metabolites, sugar beet leaf samples were extracted with KPi, as described in our previous studies [29,30]. The extraction temperature was set according to our previous trial [31] and the standard operating procedure for preparation of NMR extracts of the National Centre for Plant and Microbial Metabolomics at Rothamsted Research [32]. Briefly, 10 mg of dried leaf powder was suspended in 700 µL of KPi composed of 100 mM K_2_HPO_4_/KH_2_PO_4_ (pH or pD 7.0) in D_2_O (99.9% D; Cambridge Isotope Laboratories, Andover, MA, USA) containing 1.0 mM 2,2-dimethyl-2-silapentane-5-sulfonate sodium salt (DSS; Sigma-Aldrich, St. Louis, MI, USA). The mixture was heated at 90 °C for 5 min while shaking at 1400 rpm in a Thermomixer Comfort (Eppendorf AG, Hamburg, Germany). The KPi extract was then centrifuged (21,500× *g* for 5 min) at room temperature, and the supernatant was transferred into a 5.0-mm-diameter NMR tube (Norell, Landisville, NJ, USA) through a simple surgical cotton filter. The samples were immediately subjected to NMR measurements.

### 4.2. NMR Analysis

#### 4.2.1. NMR Spectral Measurements

NMR spectra were acquired by using an Avance-500 spectrometer (Bruker, Karlsruhe, Germany) equipped with a cryogenic probe that fits 5-mm-diameter NMR tubes (CPDUL). ^1^H-NMR spectra were collected by using the Bruker pulse program, zgpr, which uses solvent pre-saturation to remove the residual-water signal. The acquisition was done in acquisition mode with a spectral width of 20 ppm, in digital quadrature detection, with a proton 90° pulse value of 15 μs, an offset frequency of 4.7 ppm, a 4 s relaxation delay, a 20 s increment delay, 65,536 data points, and 128 scans. Two-dimensional NMR spectra were recorded on an Avance-800 spectrometer with a cryogenic probe that fits 5-mm-diameter NMR tubes (CPTCI). ^1^H-^13^C HSQC spectra were collected by using echo-anti-echo gradient selection (from the hsqcetgpsisp pulse program in the Bruker library) with 90° pulse values of 15 µs for protons and 15 µs for carbon, a 75 (f1) and 4.7 (f2) ppm offset frequency, a 2 s relaxation delay, a 160 (f1) and 12 (f2) ppm spectral width, 512 (f1) and 1,024 (f2) data points and 64 scans. When appropriate, to support metabolite annotation with the HSQC spectrum, other 2D NMR spectra (^1^H-^1^H DQF-COSY, TOCSY) were also recorded with the same instrument. The chemical shifts were calibrated by taking the signal of the DSS methyl group to be 0.00 ppm for ^1^H and ^13^C.

#### 4.2.2. Metabolite Annotation

Metabolite signals were annotated primarily by using the SpinAssign program from the PRIMe web service [33,34,35]. HSQC data were processed and the peak table was prepared by using NMRPipe and NMRDraw [36], as described previously [30]. The Human Metabolome Database (HMDB) [37,38] and the Biological Magnetic Resonance Data Bank (BMRB) [39,40] were also used. Additional metabolites in the ^1^H-NMR spectra were assigned by Chenomx Inc. (http://www.chenomx.com/).

### 4.3. ^1^H NMR-Based Metabolic Profiling

#### 4.3.1. Dataset Preparation

The data processing and the preparation of the bucket table for the ^1^H-NMR spectra were performed using TopSpin (ver. 3.2, Bruker) and Amix (ver. 3.9.14, Bruker), respectively. The AMIX underground removal tool was applied to the NMR spectra to correct the baseline before spectral binning (filter width = 40 Hz). The spectra were subdivided into integral bin areas (buckets) of 0.04 ppm over the range 10.00–0.02 ppm and the buckets were normalised to the intensity of the DSS trimethylsilyl signal at 0 ppm. Four buckets of the residual solvent signal (in the range 4.88–4.72 ppm) and ten buckets of DSS signals (in the ranges 2.96–2.88, 1.72–1.68, 0.68–0.48, and 0.02 to −0.02 ppm) were excluded from the dataset, and the resulting 237 NMR variables were used for metabolic profiling. The ranges of each bucket were 10.0–9.96, 9.96–9.92, …, and 0.02 to −0.02, and the bucketing caused no misalignments that affected the interpretation of loadings. 

#### 4.3.2. Multivariate Analysis

PCA, PLS, and OPLS were performed using SIMCA (ver. 14.1; Umetrics, Umeå, Sweden). Pareto or unit variance scaling was applied to the NMR integrals (variable *X*), and mean centring was applied to the DSI values (variable *Y*). The models were evaluated by the default seven-fold internal cross-validation, and the number of optimal components was automatically determined by the software. Models were tested for validity by 500-fold permutation tests, in which the *Y* variables (DSIs at four time points) were randomly assigned. 

#### 4.3.3. Significance Test

Welch’s *t*-test was performed on MultiExperiment Viewer (MeV) version 4.9 [41]. The significance for which any particular NMR bucket can distinguish between groups is represented by a *p*-value, where this is calculated using the *t*-distribution. No false discovery correction methods were applied to the analysis. Tukey’s multiple comparison test was performed on R software ver. 3.2.0 [42].

## Figures and Tables

**Table 1:** Sugar beet cultivars or lines used in the present study.

Cultivars/Line (Abbreviations) ^1^	Supplier ^1^	Known Resistance Level to CLS	Disease Severity Indices in 2015 ^2,3^			
			3 August	11 August	17 August	24 August
Ezomaru (Ez)	KS	Weak	3.0 ^a^	3.8 ^a^	4.6 ^a^	5.0 ^a^
Abend (Ab)	SH	Weak	2.8 ^a^	3.7 ^a,b^	4.5 ^a^	4.9 ^a^
Kachimaru (Ka)	KS	Weak	2.8 ^a,b^	3.6 ^a,b^	4.4 ^a^	4.9 ^a^
Lemiel (Le)	SH	Weak	2.8 ^a,b^	3.7 ^a,b^	4.2 ^a^	5.0 ^a^
Monohikari (Mo)	HS	Moderate	2.0 ^b^	3.1 ^b^	3.5 ^b^	4.1 ^b^
Angy (An)	SS	Strong	1.4 ^c,d^	2.4 ^c^	3.0 ^b,c^	3.8 ^b,c^
La terre (La)	SH	Strong	1.6 ^c,d^	2.4 ^c^	2.9 ^b,c,d^	3.6 ^b,c^
Hokkaimitsuboshi (Ho)	HC	Strong	1.4 ^c,d^	2.2 ^c^	2.8 ^c,d^	3.7 ^c^
Krister (Kr)	SS	Strong	1.3 ^c,d^	2.2 ^c,d^	2.7 ^c,d^	3.8 ^c^
Stout (St)	SH	Strong	1.4 ^c,d,e^	2.1 ^c,d,e^	2.5 ^c,d,e^	3.1 ^c^
Rivolta (Ri)	SS	Strong	0.8 ^d,e^	1.6 ^d,e^	2.3 ^d,e^	2.8 ^c,d^
NK-310mm-O (NK) [6]	HS	Strong	0.7 ^e^	1.5 ^e^	2.0 ^e^	2.6 ^d^

^1^ KS: KWS SAAT AG, Einbeck, Germany; SH: SESVanderHave, Tienen, Belgium; SS: Syngenta Seeds AB, Landskrona, Sweden; HC: Developed by Hokkaido Agricultural Research Centre; HS: Bred in joint breeding programs between Hokkaido Agricultural Research Centre and Syngenta Seeds AB; ^2^ For each investigation time point, data were averaged across four field block replications; ^3^ The various letters (a–e) superscripting the DSI values indicate statistically significant differences obtained by using Tukey’s multiple comparison test (*p*-value < 0.05) [20].

**Table 2:** The NMR buckets strongly contributing to discriminating CLS resistance in sugar beet leaves.

	Bucket (ppm)	Presumed Compounds	PLS-VIP				Pearson’s Correlation for DSIs on August				Welch’s *t*-Test *p*-Value
			*Y* Variable = DSIs on August								
			4	11	17	24	4	11	17	24	
Seedling stage	3.96–3.92	Unidentified	2.46	2.40	2.32	2.51	0.55	0.52	0.52	0.52	0.00404
Early growth stage	3.00–2.96	GABA, Asn	2.52	2.55	2.44	2.60	−0.70	−0.69	−0.66	−0.69	0.00011
	2.36–2.32	Glu, MA	2.33	2.33	2.22	2.24	−0.65	−0.63	−0.60	−0.60	0.00068
	2.44–2.40	Gln	2.26	2.36	2.27	2.45	−0.63	−0.63	−0.61	−0.65	0.00266
	8.00–7.96	Unidentified	2.13	2.04	2.09	*	0.59	0.55	0.56	0.52	0.00048
	2.32–2.28	GABA, Glu, Pro	2.12	2.13	2.17	2.15	−0.59	−0.57	−0.59	−0.57	0.00048
	2.28–2.24	GABA, Val	2.11	2.07	2.15	2.06	−0.59	−0.55	−0.58	−0.55	0.00016
	5.04–5.00	Unidentified	2.10	2.06	2.13	2.01	0.58	0.55	0.57	0.54	0.00046
	1.92–1.88	GABA, Acetate	2.07	2.07	2.12	2.02	−0.58	−0.57	−0.55	−0.54	0.00356
	5.72–5.68	Unidentified	2.07	2.07	2.18	2.37	−0.57	−0.58	−0.56	−0.63	0.00500
	2.12–2.08	Glu/Gln/Pro	1.83	1.92	1.94	1.92	−0.51	−0.52	−0.52	−0.51	0.00762
Root-enlargement stage	5.04–5.00	Unidentified	2.79	2.75	2.79	2.63	0.66	0.63	0.65	0.59	0.00003
	8.00–7.96	Unidentified	2.40	2.34	2.39	2.30	0.57	0.53	0.56	0.52	0.00075
	2.44–2.40	Gln	2.19	2.31	2.31	2.29	−0.52	−0.53	−0.53	−0.52	0.01508
	5.72–5.68	Unidentified	2.18	2.40	2.24	2.63	−0.52	−0.55	−0.52	−0.59	0.01293
Disease-development stage	3.96–3.92	Unidentified	3.05	2.94	3.02	2.78	0.64	0.53	0.64	0.58	0.00014
	8.00–7.96	Unidentified	2.79	2.59	2.75	2.58	0.59	0.51	0.58	0.54	0.00199
	3.60–3.56	Suc, Fru	2.35	2.46	2.47	2.41	0.54	0.53	0.56	0.56	0.01193

## Supplementary Materials

The following are available online at www.mdpi.com/2218-1989/7/1/4/s1, Figure S1: Daily values of mean air temperature, mean relative humidity, and rainfall during the field trial in 2015 (Memuro, Hokkaido, Japan; 42°89.2′N/143°0.7.7′E, 92 m a.s.l.); Figure S2: Metabolite annotation of representative ^1^H-NMR spectra of sugar beet leaf samples; Figure S3: PCs 3 and 4 plane of the PCA score (A,C) and loading (B,D) plots of NMR data of sugar beet leaves; Figure S4: Expanded views of an aromatic region of ^1^H-^13^C HSQC (A), ^1^H-^1^H DQF-COSY (B), and ^1^H-^1^H TOCSY (C) spectra of Ez-2 (blue) and NK-1 (red) at the seedling stage; Figure S5: Relative intensity of the isolated NMR buckets annotated to GABA (1.882–1.856 ppm) and Gln (2.470–2.440 ppm) at the early growth stage (A,B), Gln at the root-enlargement stage (C), and Fru (4.110–4.101 ppm) at the disease development stage (D); Table S1: PLS model parameters derived from the ^1^H-NMR spectra of sugar beet leaves. DSIs recorded at four time points were separately used as *Y* variables; Table S2: OPLS model parameters derived from the ^1^H-NMR spectra of sugar beet leaves. DSIs recorded at four time points were separately used as *Y* variables; Table S3: The number of permuted *R*^2^ and *Q*^2^ values exceeding the original values for PLS models; Table S4: The number of permuted *R*^2^ and *Q*^2^ values exceeding the original values for OPLS models; Table S5: NMR buckets with the highest PLS VIP values (rank 1–20) at the seedling stage; Table S6: NMR buckets with the highest PLS VIP values (rank 1–20) at the early growth stage; Table S7: NMR buckets with the highest PLS VIP values (rank 1–20) at the root-enlargement stage; Table S8: NMR buckets with the highest PLS VIP values (rank 1–20) at the disease-development stage; Table S9: NMR buckets with the highest OPLS VIP values (rank 1–20) at the seedling stage; Table S10: NMR buckets with the highest OPLS VIP values (rank 1–20) at the early growth stage; Table S11: NMR buckets with the highest OPLS VIP values (rank 1–20) at the root-enlargement stage; Table S12: NMR buckets with the highest OPLS VIP values (rank 1–20) at the disease-development stage; Table S13: Pearson’s correlation coefficient between the original intensity of NMR buckets (normalized to the intensity of DSS) and DSIs. The buckets with |*r*| > 0.5 are listed; Table S14: The NMR buckets strongly contributing to discriminating CLS resistance in sugar beet leaves.
