# Supplementary material for: NMR-Based Metabolic Profiling of Field-Grown Leaves from Sugar Beet Plants Harbouring Different Levels of Resistance to Cercospora Leaf Spot Disease
Source: Metabolites. 2017 Jan 26;7(1):4. doi: 10.3390/metabo7010004 (PMC5372207; doi:10.3390/metabo7010004)
Supplement: Supplementary file 1 [file metabolites-07-00004-s001.pdf]

Supplementary Materials: NMR-Based Metabolic Profiling of Field-Grown Leaves from Sugar Beet Plants Harbouring Different Levels of Resistance to *Cercospora* Leaf Spot Disease

Yasuyo Sekiyama, Kazuyuki Okazaki, Jun Kikuchi and Seishi Ikeda

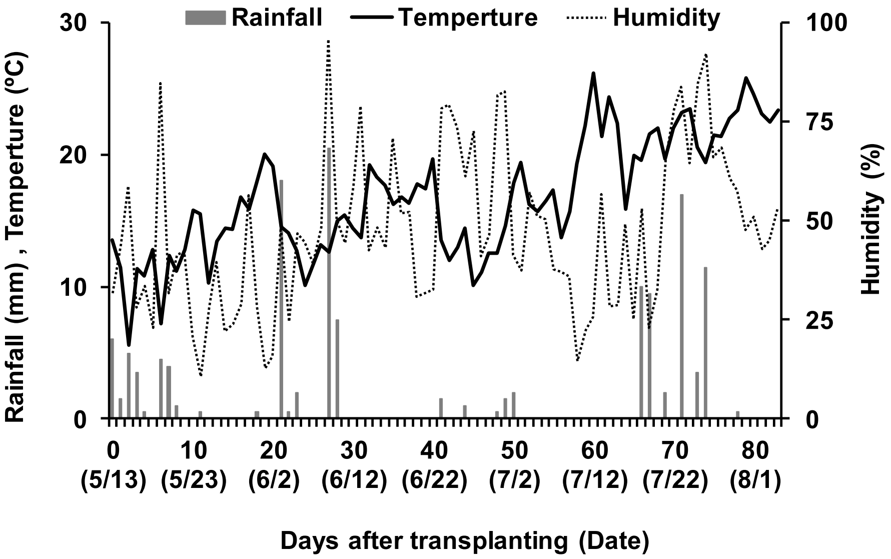

Figure S1. Daily values of mean air temperature, mean relative humidity, and rainfall during the field trial in 2015 (Memuro, Hokkaido, Japan; 42°89.2′N/143°0.7.7′E, 92 m a.s.l.).

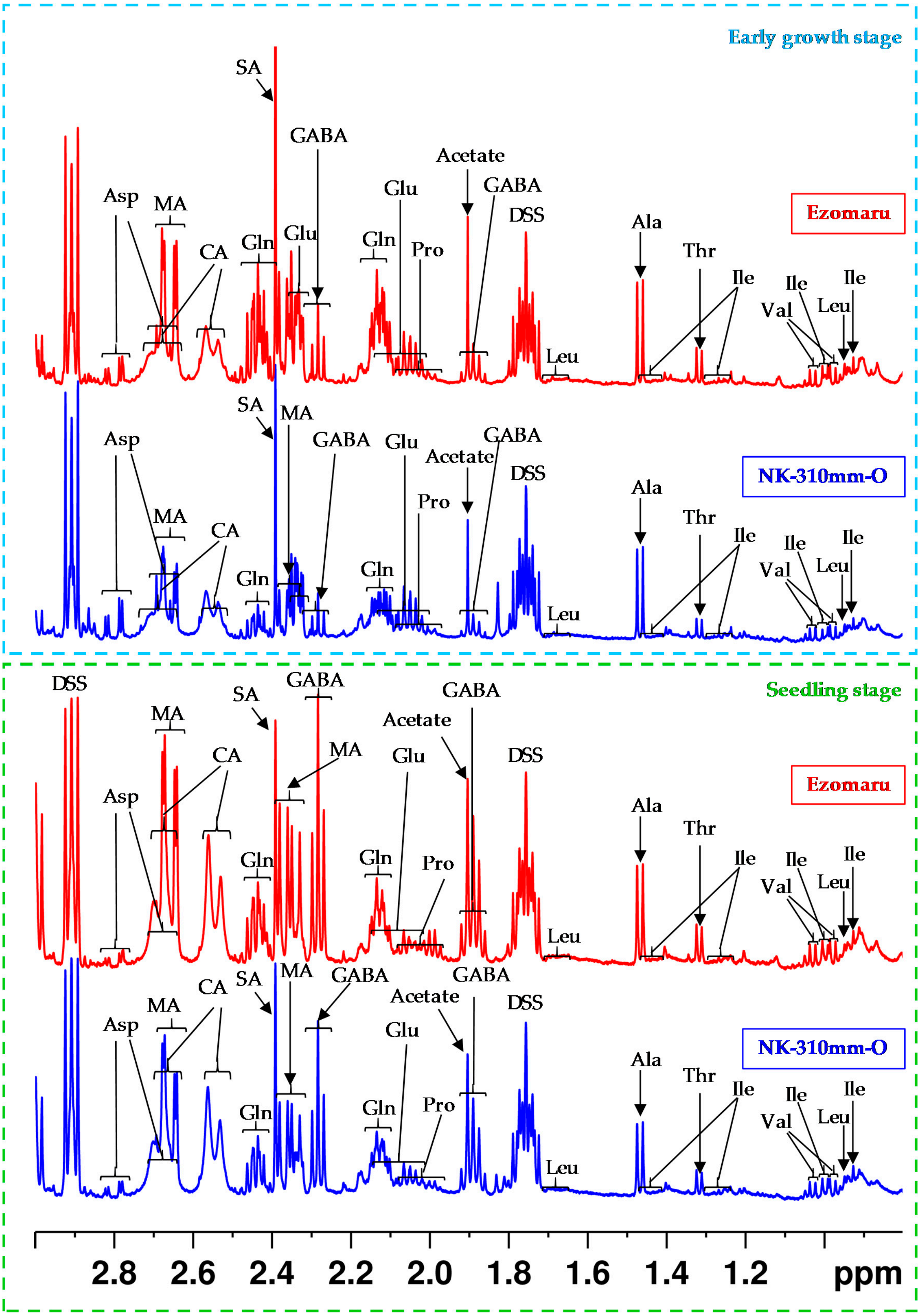

Figure S2. *Cont.*

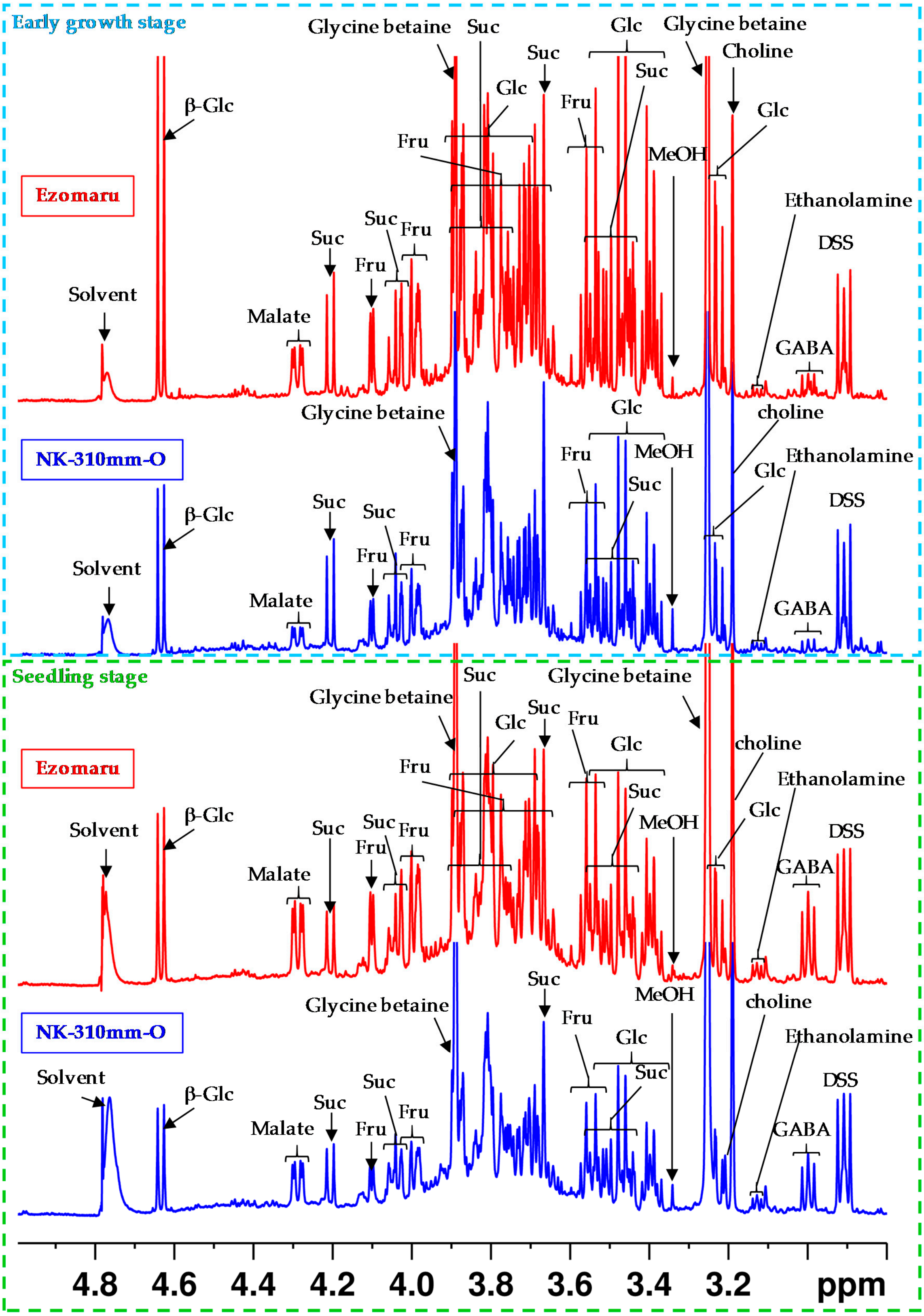

Figure S2. *Cont.*

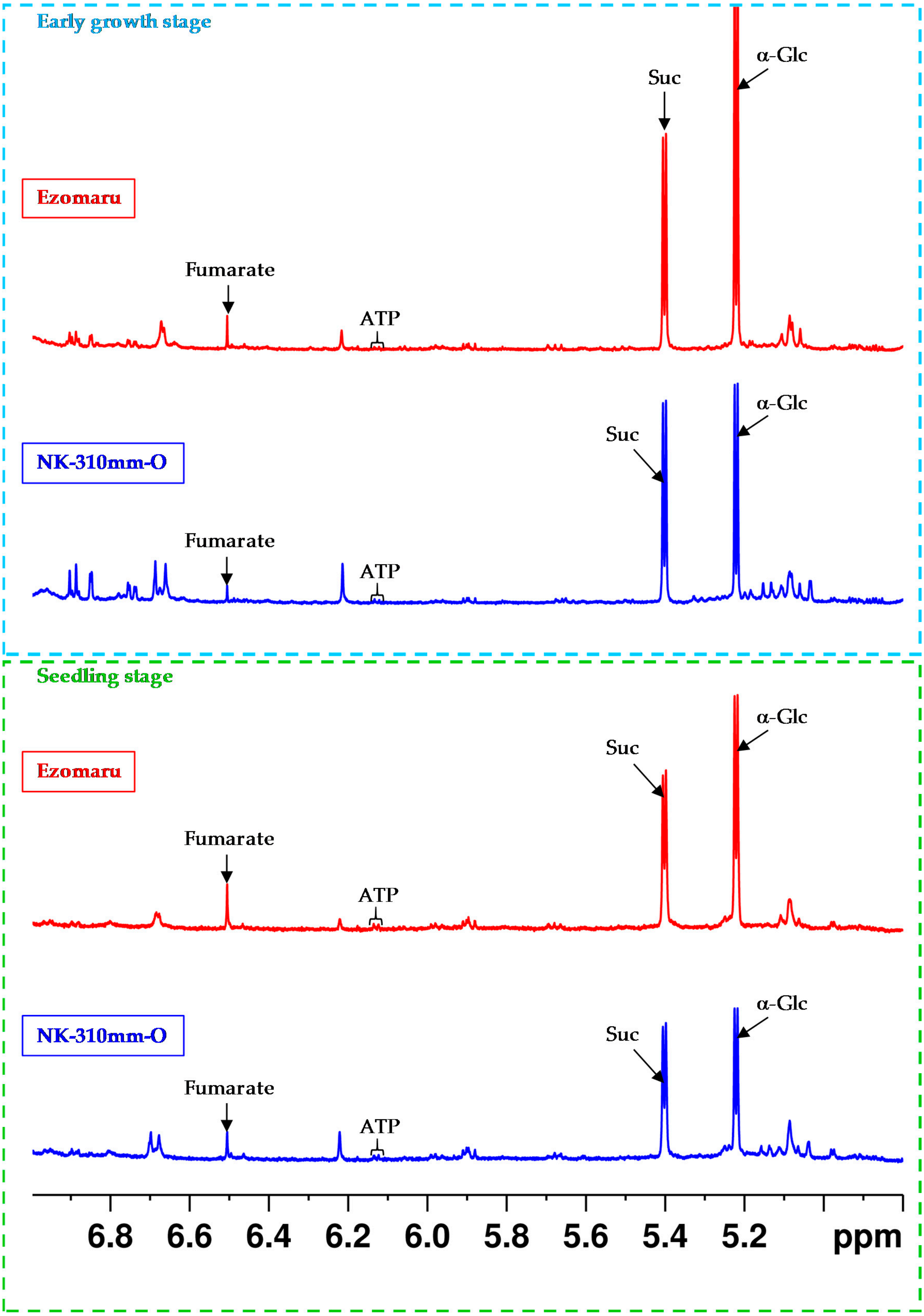

Figure S2. *Cont.*

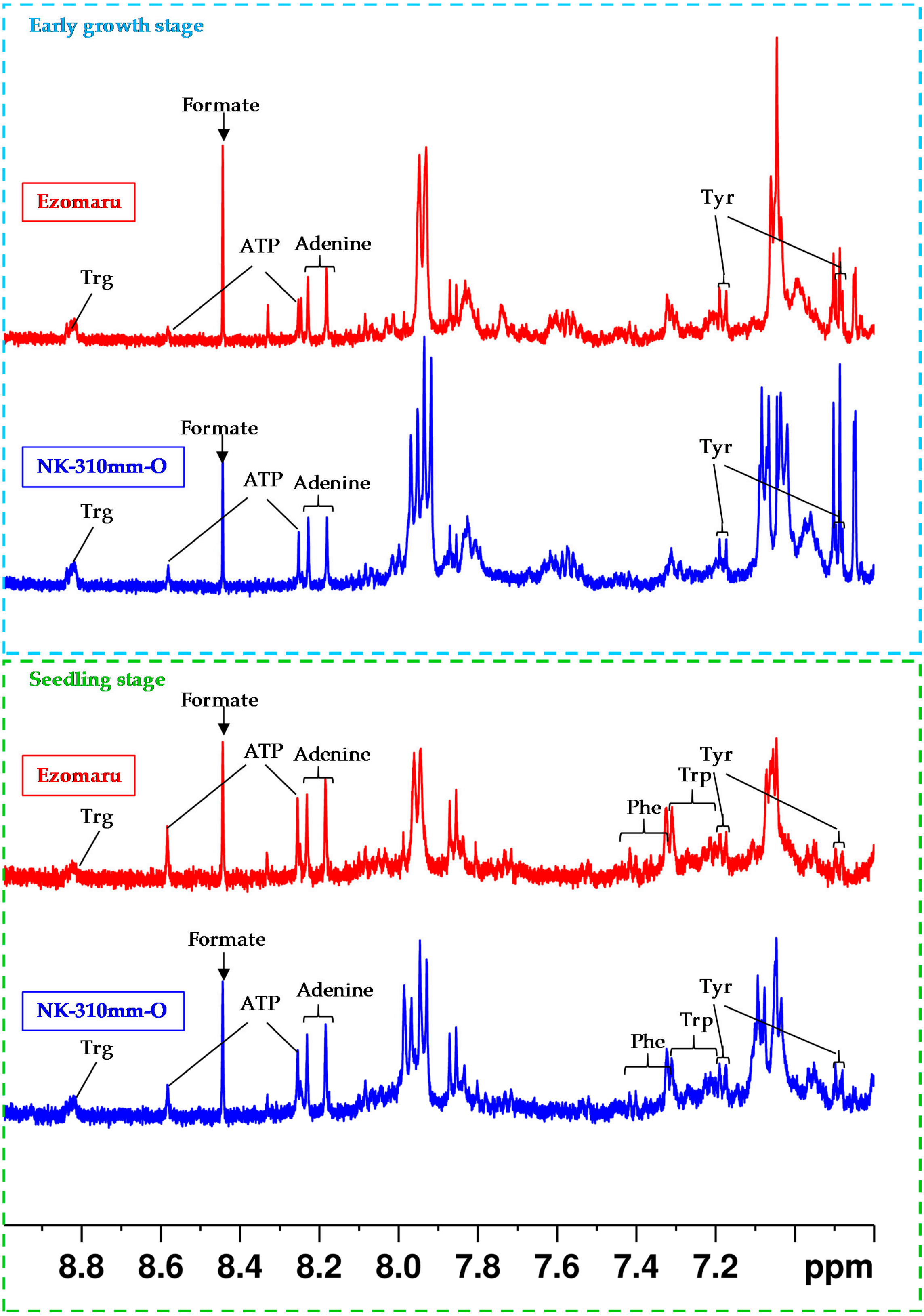

Figure S2. Metabolite annotation of representative ^{1}H-NMR spectra of sugar beet leaf samples. The spectra of Ezomaru (red line) and at the seedling stage represented by NK-310mm-O (blue line) are shown in the light-green dashed-line rectangle (bottom). Those at an early growth stage are shown in the light-blue dashed-line rectangle (top).

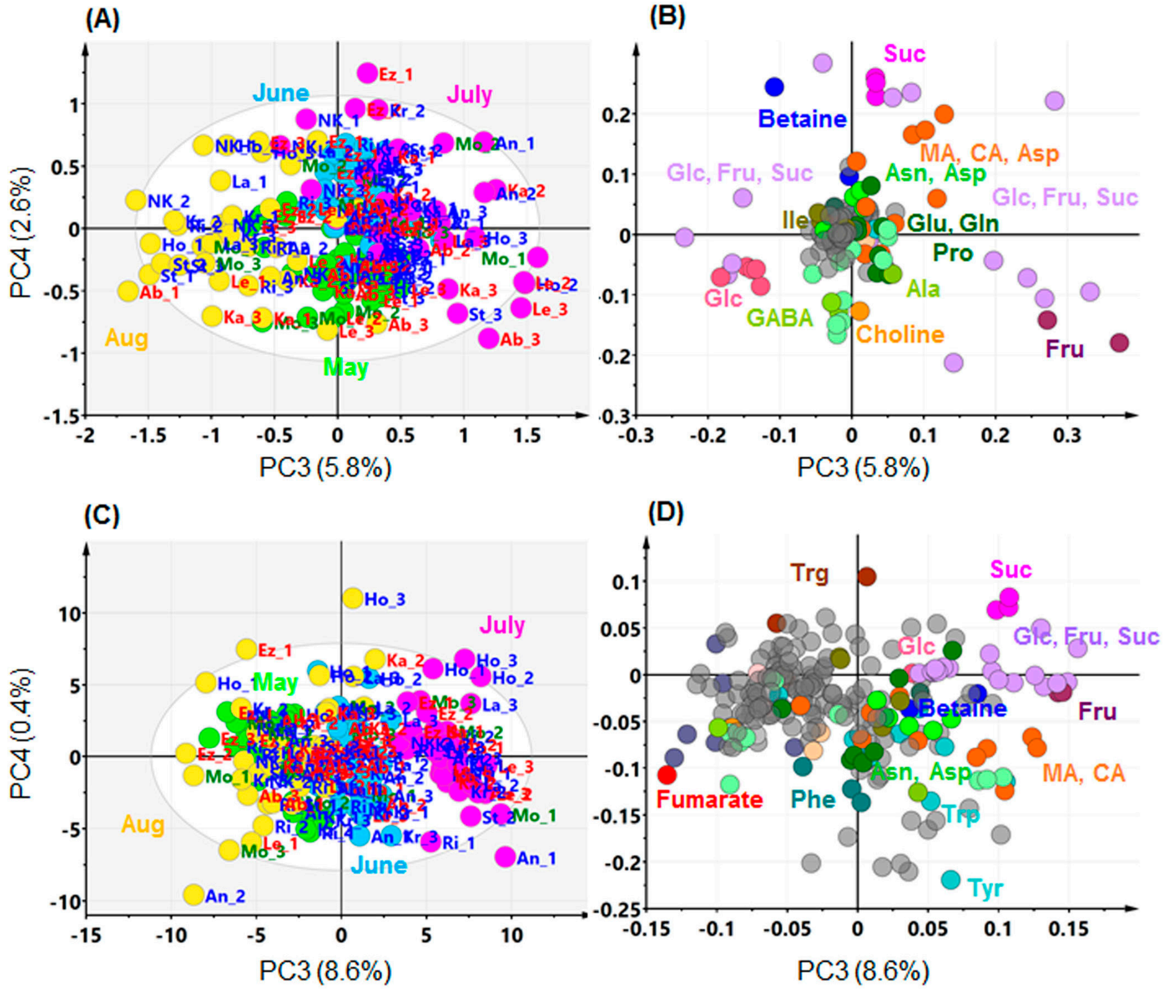

Figure S3. PCs 3 and 4 plane of the PCA score (**A**,**C**) and loading (**B**,**D**) plots of NMR data of sugar beet leaves. Pareto (**A**,**B**) or unit variance (**C**,**D**) scaling was applied to the NMR buckets. The abbreviations of genotypes harbouring weak (red), moderate (green), and strong (blue) resistance levels are defined in Table 1. Grey circles in the loading plots indicate Unannotated NMR buckets.

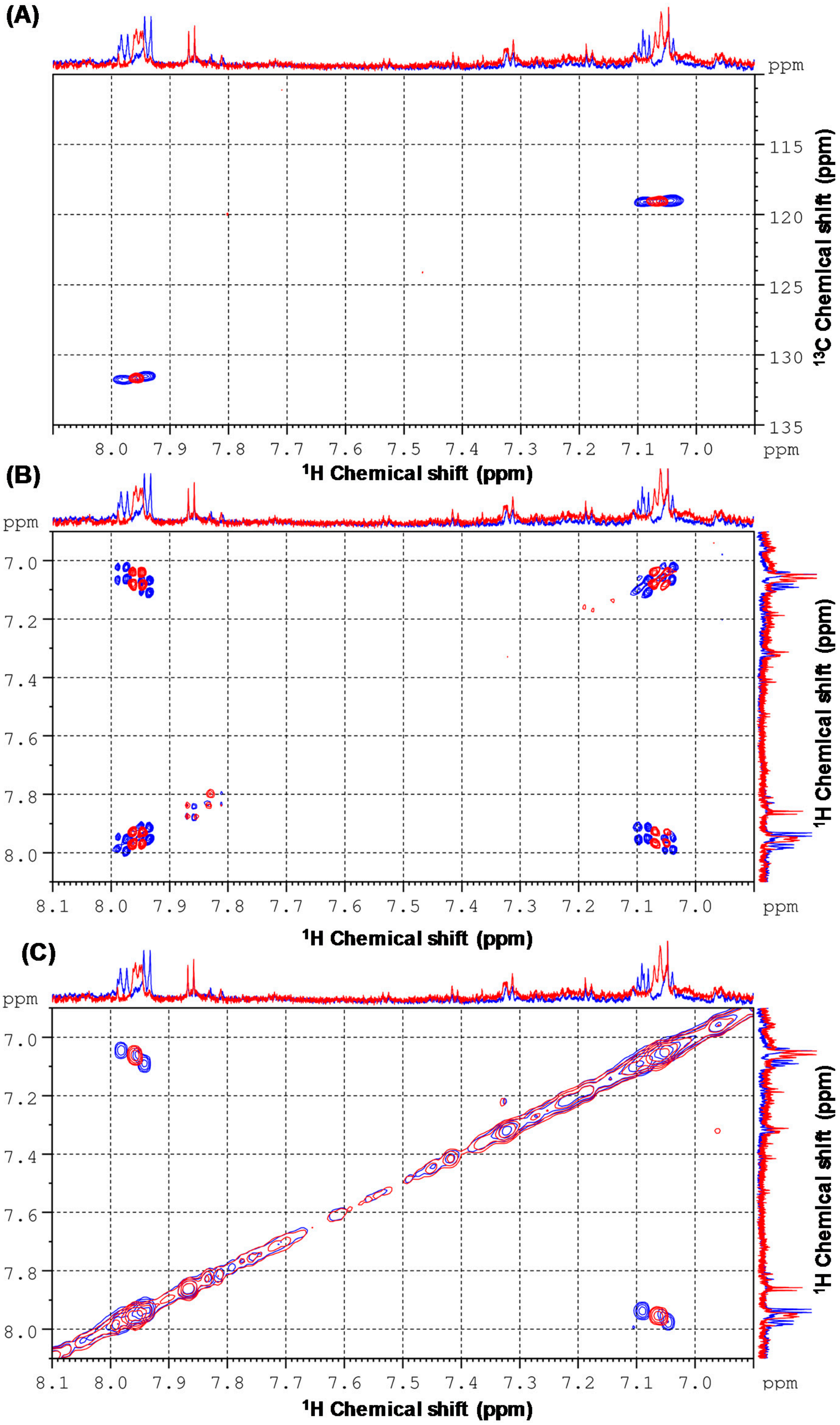

Figure S4. Expanded views of an aromatic region of ^{1}H-^{13}C HSQC (**A**), ^{1}H-^{1}H DQF-COSY (**B**), and ^{1}H-^{1}H TOCSY (C) spectra of Ez-2 (red) and NK-1 (blue) at the seedling stage.

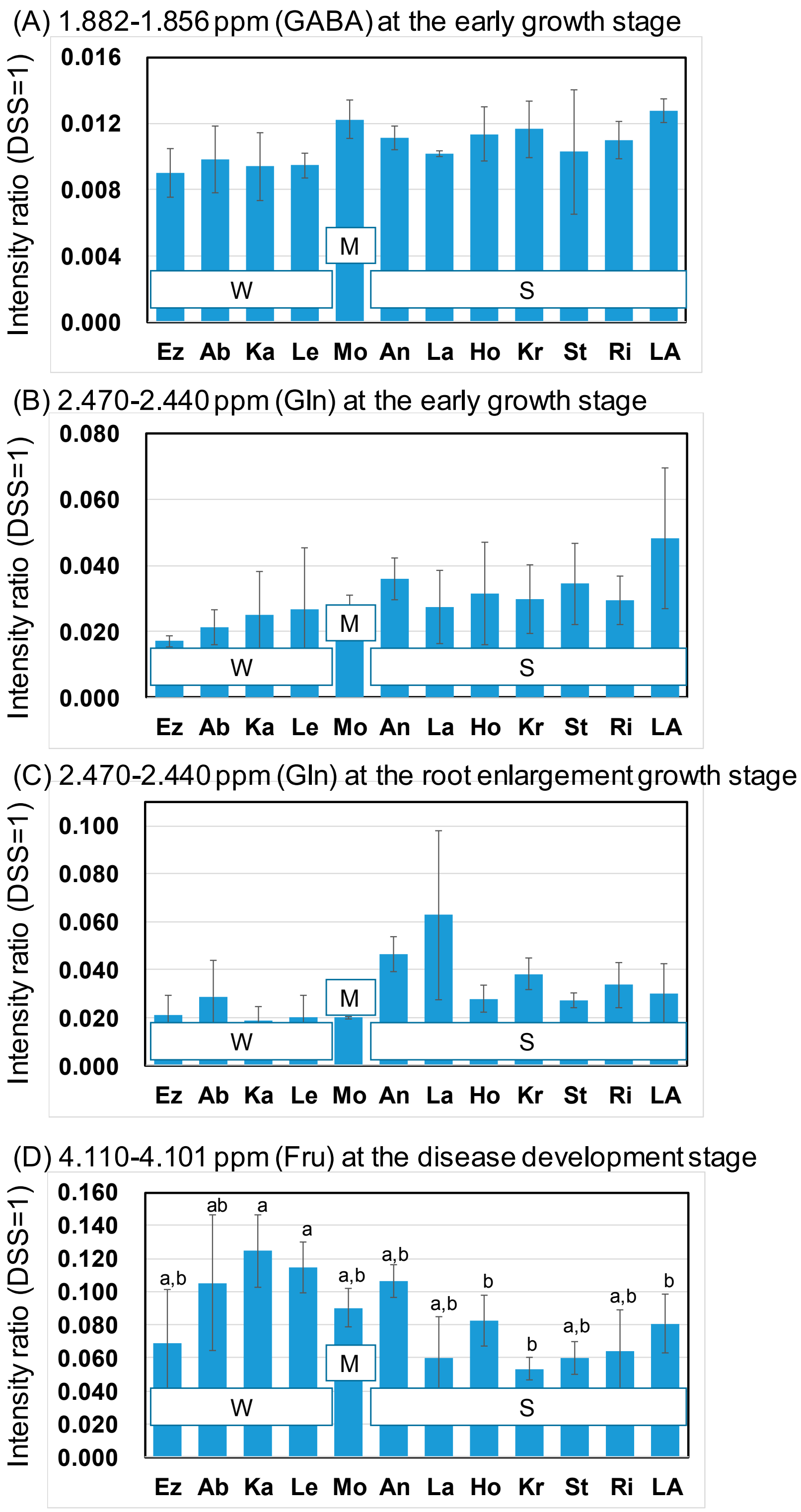

Figure S5. Relative intensity of the isolated NMR buckets annotated to GABA (1.882–1.856 ppm) and Gln (2.470–2.440 ppm) at the early growth stage (**A**,**B**), Gln at the root-enlargement stage (**C**), and Fru (4.110–4.101 ppm) at the disease development stage (**D**). Letters a and b superscripting the error bars indicate statistically significant differences among genotypes obtained using Tukey's multiple comparison test ($p < 0.05$). The known resistance levels are indicated in white rectangles: W represents genotypes with weak resistance, M represents genotypes with moderate resistance, and S represents genotypes with strong resistance. No significant difference with the p-values less than 0.05 was observed in the intensity of GABA and Gln among genotypes.

Table S1. PLS model parameters derived from the ^{1}H-NMR spectra of sugar beet leaves. DSIs recorded at four time points were separately used as *Y* variables.

Growth Stage (Sampling Date)	DSIs on	Pareto Scaling			Unit Variance Scaling		
		NC [1]	R^2 (Intercept) [2]	Q^2 (Intercept) [2]	NC [1]	R^2 (Intercept) [2]	Q^2 (Intercept) [2]
Seedling stage (11 May)	3 August	0			1	0.79 (0.43)	0.4 (−0.12)
	11 August	0			1	0.76 (0.43)	0.32 (−0.12)
	17 August	0			2	0.83 (0.72)	0.41 (−0.09)
	24 August	0			1	0.78 (0.43)	0.26 (−0.11)
Early growth stage (18 July)	3 August	3	0.66 (0.36)	0.45 (−0.20)	1	0.71 (0.53)	0.51 (−0.15)
	11 August	3	0.62 (0.36)	0.36 (−0.21)	1	0.67 (0.53)	0.45 (−0.14)
	17 August	3	0.62 (0.37)	0.36 (−0.19)	1	0.66 (0.54)	0.47 (−0.14)
	24 August	3	0.6 (0.37)	0.33 (−0.19)	1	0.65 (0.53)	0.43 (−0.12)
Root-enlargement stage (13 July)	3 August	3	0.53 (0.29)	0.20 (−0.23)	1	0.61 (0.53)	0.24 (−0.11)
	11 August	1	0.28 (0.07)	0.06 (−0.07)	1	0.58 (0.53)	0.18 (−0.11)
	17 August	2	0.38 (0.19)	0.17 (−0.14)	1	0.59 (0.53)	0.22 (−0.11)
	24 August	3	0.40 (0.20)	0.19 (−0.14)	1	0.57 (0.53)	0.15 (−0.10)
Disease development stage (4 August)	3 August	0			1	0.78 (0.54)	0.4 (−0.11)
	11 August	0			1	0.79 (0.55)	0.37 (−0.12)
	17 August	0			1	0.78 (0.55)	0.32 (−0.11)
	24 August	0			1	0.75 (0.55)	0.31 (−0.12)

[1] NC: Number of components; [2] Intercept of the permutation plot (n = 500) where the vertical axis corresponds to R^2 and Q^2 values of each model and the horizontal axis corresponds to the correlation coefficient between the original *Y* and the permuted *Y*.

Table S2. OPLS model parameters derived from the ^{1}H-NMR spectra of sugar beet leaves. DSIs recorded at four time points were separately used as *Y* variables.

Growth Stage (Sampling Date)	DSIs on	Pareto Scaling			Unit Variance Scaling		
		NC [1]	R^2 (Intercept) [2]	Q^2 (Intercept) [2]	NC [1]	R^2 (Intercept) [2]	Q^2 (Intercept) [2]
Seedling stage (11 May)	3 August	0 + 0			1 + 1	0.87 (0.73)	0.53 (−0.39)
	11 August	0 + 0			1 + 1	0.85 (0.73)	0.46 (−0.38)
	17 August	0 + 0			1 + 1	0.84 (0.73)	0.44 (−0.38)
	24 August	0 + 0			1 + 1	0.91 (0.72)	0.40 (−0.38)
Early growth stage (18 July)	3 August	1 + 2	0.66 (0.37)	0.43 (−0.44)	1 + 2	0.95 (0.90)	0.57 (−0.44)
	11 August	1 + 2	0.62 (0.37)	0.35 (−0.43)	1 + 2	0.95 (0.90)	0.52 (−0.42)
	17 August	1 + 2	0.62 (0.36)	0.36 (−0.45)	1 + 2	0.95 (0.78)	0.57 (−0.38)
	24 August	1 + 2	0.60 (0.37)	0.34 (−0.44)	1 + 0	0.65 (0.53)	0.43 (−0.32)
Root-enlargement stage (13 July)	3 August	1 + 2	0.53 (0.29)	0.31 (−0.44)	1 + 0	0.61 (0.52)	0.24 (−0.32)
	11 August	1 + 2	0.51 (0.30)	0.28 (−0.46)	1 + 0	0.58 (0.53)	0.18 (−0.32)
	17 August	1 + 2	0.52 (0.30)	0.30 (−0.42)	1 + 0	0.59 (0.53)	0.22 (−0.31)
	24 August	1 + 2	0.51 (0.30)	0.29 (−0.44)	1 + 0	0.57 (0.53)	0.15 (−0.31)
Disease development stage (4 August)	3 August	1 + 1	0.48 (0.18)	0.19 (−0.28)	1 + 1	0.89 (0.81)	0.45 (−0.34)
	11 August	1 + 1	0.48 (0.18)	0.20 (−0.31)	1 + 1	0.90 (0.81)	0.50 (−0.34)
	17 August	1 + 1	0.52 (0.17)	0.19 (−0.30)	1 + 1	0.89 (0.81)	0.46 (−0.35)
	24 August	1 + 4	0.83 (0.64)	0.43 (−0.77)	1 + 1	0.86 (0.81)	0.45 (−0.37)

[1] NC: Number of components; [2] Intercept of the permutation plot (n = 500) where the vertical axis corresponds to R^2 and Q^2 values of each model and the horizontal axis corresponds to the correlation coefficient between the original *Y* and the permuted *Y*.

Table S3. The number of permuted R^2 and Q^2 values exceeding the original values for PLS models.

Growth Stage (Sampling Date)	DSIs on	Pareto Scaling		Unit Variance Scaling	
		R^2	Q^2	R^2	Q^2
Seedling stage (11 May)	3 August			4	0
	11 August			8	0
	17 August			18	0
	24 August			0	1
Early growth stage (18 July)	3 August	0	0	16	1
	11 August	2	0	15	0
	17 August	1	1	21	0
	24 August	3	1	42	0
Root-enlargement stage (13 July)	3 August	9	6	80	0
	11 August	4	37	132	11
	17 August	20	12	109	3
	24 August	9	6	141	11
Disease development stage (4 August)	3 August			25	0
	11 August			15	0
	17 August			22	0
	24 August			79	0

Table S4. The number of permuted R^2 and Q^2 values exceeding the original values for OPLS models.

Growth Stage (Sampling Date)	DSIs on	Pareto Scaling		Unit Variance Scaling	
		R^2	Q^2	R^2	Q^2
Seedling stage (11 May)	3 August			0	0
	11 August			0	1
	17 August			18	1
	24 August			0	2
Early growth stage (18 July)	3 August	1	0	15	0
	11 August	2	1	39	0
	17 August	1	0	58	0
	24 August	4	2	66	0
Root-enlargement stage (13 July)	3 August	11	1	78	4
	11 August	9	1	130	6
	17 August	9	1	117	1
	24 August	7	0	155	15
Disease development stage (4 August)	3 August	3	5	1	0
	11 August	2	5	2	0
	17 August	0	1	4	0
	24 August	2	0	9	0

Table S5. NMR buckets with the highest PLS VIP values (rank 1–20) at the seedling stage.

	Y Variable = DSIs at 3 August			*Y* Variable = DSIs at 11 August			*Y* Variable = DSIs at 17 August			*Y* Variable = DSIs at 24 August		
	**Bucket (ppm)**	**Presumed Compounds**	**VIP**	**Bucket (ppm)**	**Presumed Compounds**	**VIP**	**Bucket (ppm)**	**Presumed Compounds**	**VIP**	**Bucket (ppm)**	**Presumed Compounds**	**VIP**
Seedling stage	6.24–6.20	Unannotated	2.47	9.28–9.24	Unannotated	2.44	9.28–9.24	Unannotated	2.37	5.04–5.00	Unannotated	2.63
	5.04–5.00	Unannotated	2.46	6.24–6.20	Unannotated	2.41	3.96–3.92	Unannotated	2.32	0.16–0.12	Unannotated	2.53
	3.96–3.92	Unannotated	2.46	3.96–3.92	Unannotated	2.40	6.76–6.72	Unannotated	2.29	3.96–3.92	Unannotated	2.51
	9.28–9.24	Unannotated	2.40	6.16–6.12	ATP	2.39	5.04–5.00	Unannotated	2.29	5.96–5.92	Unannotated	2.45
	6.16–6.12	ATP	2.39	5.04–5.00	Unannotated	2.30	6.24–6.20	Unannotated	2.24	9.28–9.24	Unannotated	2.41
	6.76–6.72	Unannotated	2.31	0.16–0.12	Unannotated	2.27	1.32–1.28	Thr	2.09	6.24–6.20	Unannotated	2.40
	8.00–7.96	Unannotated	2.30	8.84–8.80	Trg	2.22	8.00–7.96	Unannotated	2.08	8.00–7.96	Unannotated	2.33
	0.16–0.12	Unannotated	2.20	6.76–6.72	Unannotated	2.22	0.16–0.12	Unannotated	2.01	5.08–5.04	Unannotated	2.26
	1.32–1.28	Thr	2.12	5.96–5.92	Unannotated	2.21	8.84–8.80	Trg	2.00	6.76–6.72	Unannotated	2.24
	8.84–8.80	Trg	2.12	8.60–8.56	ATP	2.20	6.16–6.12	ATP	1.94	5.72–5.68	Unannotated	2.23
	8.60–8.56	ATP	2.09	1.32–1.28	Thr	2.13	1.84–1.80	Unannotated	1.88	6.16–6.12	ATP	2.23
	5.96–5.92	Unannotated	2.09	8.00–7.96	Unannotated	2.12	4.28–4.24	MA/Thr	1.88	1.32–1.28	Thr	2.14
	1.84–1.80	Unannotated	1.97	4.28–4.24	MA/Thr	2.01	0.72–0.68	Unannotated	1.86	0.72–0.68	Unannotated	2.12
	6.96–6.92	Unannotated	1.96	5.72–5.68	Unannotated	1.98	5.96–5.92	Unannotated	1.85	8.60–8.56	ATP	2.09
	0.84–0.80	Unannotated	1.92	0.72–0.68	Unannotated	1.92	2.00–1.96	Ile/Pro	1.85	8.84–8.80	Trg	2.03
	4.28–4.24	MA/Thr	1.91	1.84–1.80	Unannotated	1.89	2.44–2.40	Gln	1.85	0.84–0.80	Unannotated	1.97
	0.72–0.68	Unannotated	1.86	2.00–1.96	Ile/Pro	1.86	5.08–5.04	Unannotated	1.80	1.36–1.32	Thr	1.92
	2.00–1.96	Ile/Pro	1.86	6.96–6.92	Unannotated	1.86	2.64–2.60	Asp/MA	1.78	1.56–1.52	Unannotated	1.91
	5.08–5.04	Unannotated	1.84	0.84–0.80	Unannotated	1.84	1.36–1.32	Thr	1.77	2.00–1.96	Ile/Pro	1.84
	1.36–1.32	Thr	1.83	1.36–1.32	Thr	1.83	0.84–0.80	Unannotated	1.73	9.48–9.44	Unannotated	1.84

Table S6. NMR buckets with the highest PLS VIP values (rank 1–20) at the early growth stage.

	Y Variable = DSIs at 3 August			*Y* Variable = DSIs at 11 August			*Y* Variable = DSIs at 17 August			*Y* Variable = DSIs at 24 August		
	**Bucket (ppm)**	**Presumed Compounds**	**VIP**	**Bucket (ppm)**	**Presumed Compounds**	**VIP**	**Bucket (ppm)**	**Presumed Compounds**	**VIP**	**Bucket (ppm)**	**Presumed Compounds**	**VIP**
Early growth stage	3.00–2.96	GABA/Asn	2.52	3.00–2.96	GABA/Asn	2.55	3.00–2.96	GABA/Asn	2.44	3.00–2.96	GABA/Asn	2.60
	2.36–2.32	Glu/MA	2.33	2.44–2.40	Gln	2.36	2.44–2.40	Gln	2.27	2.44–2.40	Gln	2.45
	2.44–2.40	Gln	2.26	2.36–2.32	Glu/MA	2.33	2.36–2.32	Glu/MA	2.22	5.72–5.68	Unannotated	2.37
	8.00–7.96	Unannotated	2.13	5.72–5.68	Unannotated	2.18	2.32–2.28	GABA/Glu/Pro	2.17	2.36–2.32	Glu/MA	2.24
	2.32–2.28	GABA/Glu/Pro	2.12	2.32–2.28	GABA/Glu/Pro	2.13	2.28–2.24	GABA/Val	2.15	2.32–2.28	GABA/Glu/Pro	2.15
	2.28–2.24	GABA/Val	2.11	1.92–1.88	GABA/Acetate	2.12	5.04–5.00	Unannotated	2.13	2.28–2.24	GABA/Val	2.06
	5.04–5.00	Unannotated	2.10	2.28–2.24	GABA/Val	2.07	5.72–5.68	Unannotated	2.09	7.56–7.52	Trp	2.03
	1.92–1.88	GABA/Acetate	2.07	5.04–5.00	Unannotated	2.06	8.00–7.96	Unannotated	2.09	1.92–1.88	GABA/Acetate	2.02
	5.72–5.68	Unannotated	2.07	8.00–7.96	Unannotated	2.04	1.92–1.88	GABA/Acetate	2.03	5.04–5.00	Unannotated	2.01
	4.28–4.24	MA/Thr	1.95	2.12–2.08	Glu/Gln/Pro	1.92	3.04–3.00	GABA	1.96	8.00–7.96	Unannotated	1.95
	2.40–2.36	SA/MA	1.95	7.56–7.52	Trp	1.87	1.84–1.80	Unannotated	1.94	2.12–2.08	Glu/Gln/Pro	1.92
	6.96–6.92	Unannotated	1.89	1.84–1.80	Unannotated	1.87	2.12–2.08	Glu/Gln/Pro	1.94	1.56–1.52	Unannotated	1.87
	7.56–7.52	Trp	1.89	2.40–2.36	SA/MA	1.86	2.64–2.60	Asp/MA	1.91	2.40–2.36	SA/MA	1.80
	1.84–1.80	Unannotated	1.88	3.04–3.00	GABA	1.85	1.88–1.84	GABA	1.85	1.84–1.80	Unannotated	1.78
	2.64–2.60	Asp/MA	1.86	4.28–4.24	MA/Thr	1.83	6.36–6.32	Unannotated	1.83	1.88–1.84	GABA	1.77
	4.32–4.28	MA	1.85	1.56–1.52	Unannotated	1.83	0.96–0.92	Ile/Leu	1.82	2.16–2.12	Glu/Gln	1.76
	1.88–1.84	GABA	1.83	6.96–6.92	Unannotated	1.79	2.16–2.12	Glu/Gln	1.82	2.48–2.44	Gln	1.73
	2.12–2.08	Glu/Gln/Pro	1.83	2.64–2.60	Asp/MA	1.78	7.56–7.52	Trp	1.79	1.32–1.28	Thr	1.72
	6.36–6.32	Unannotated	1.81	1.88–1.84	GABA	1.77	6.96–6.92	Unannotated	1.79	3.04–3.00	GABA	1.72
	3.04–3.00	GABA	1.80	0.96–0.92	Ile/Leu	1.75	2.48–2.44	Gln	1.77	6.96–6.92	Unannotated	1.71

Table S7. NMR buckets with the highest PLS VIP values (rank 1–20) at the root-enlargement stage.

	***Y* Variable = DSIs at 3 August**			***Y* Variable = DSIs at 11 August**			***Y* Variable = DSIs at 17 August**			***Y* Variable = DSIs at 24 August**		
	**Bucket (ppm)**	**Presumed Compounds**	**VIP**	**Bucket (ppm)**	**Presumed Compounds**	**VIP**	**Bucket (ppm)**	**Presumed Compounds**	**VIP**	**Bucket (ppm)**	**Presumed Compounds**	**VIP**
Root-enlargement stage	5.04–5.00	Unannotated	2.79	5.04–5.00	Unannotated	2.75	5.04–5.00	Unannotated	2.79	5.04–5.00	Unannotated	2.63
	8.00–7.96	Unannotated	2.40	5.72–5.68	Unannotated	2.40	8.00–7.96	Unannotated	2.39	5.72–5.68	Unannotated	2.63
	2.44–2.40	Gln	2.19	8.00–7.96	Unannotated	2.34	6.80–6.76	Unannotated	2.29	8.00–7.96	Unannotated	2.30
	5.72–5.68	Unannotated	2.18	2.44–2.40	Gln	2.31	2.44–2.40	Gln	2.24	2.44–2.40	Gln	2.29
	6.80–6.76	Unannotated	2.10	2.12–2.08	Glu/Gln/Pro	2.18	5.72–5.68	Unannotated	2.24	3.12–3.08	Ethanolamine	2.24
	3.12–3.08	Ethanolamine	2.10	3.12–3.08	Ethanolamine	2.16	2.12–2.08	Glu/Gln/Pro	2.13	3.04–3.00	GABA	2.14
	3.04–3.00	GABA	2.09	6.80–6.76	Unannotated	2.11	2.16–2.12	Glu/Gln	2.05	2.12–2.08	Glu/Gln/Pro	2.10
	2.12–2.08	Glu/Gln/Pro	2.06	3.04–3.00	GABA	2.10	3.04–3.00	GABA	2.02	3.00–2.96	GABA/Asn	2.09
	2.16–2.12	Glu/Gln	1.99	2.16–2.12	Glu/Gln	2.08	3.12–3.08	Ethanolamine	2.02	1.36–1.32	Thr	2.07
	2.48–2.44	Gln	1.89	2.48–2.44	Gln	1.97	2.48–2.44	Gln	1.95	2.16–2.12	Glu/Gln	2.01
	1.84–1.80	Unannotated	1.87	2.36–2.32	Glu/MA	1.86	1.84–1.80	Unannotated	1.95	6.80–6.76	Unannotated	1.97
	1.28–1.24	Ile	1.85	1.28–1.24	Ile	1.85	2.36–2.32	Glu/MA	1.87	2.32–2.28	GABA/Glu/Pro	1.94
	2.36–2.32	Glu/MA	1.84	1.84–1.80	Unannotated	1.83	1.28–1.24	Ile	1.83	1.28–1.24	Ile	1.93
	6.88–6.84	Tyr	1.84	3.00–2.96	GABA/Asn	1.83	6.88–6.84	Tyr	1.80	2.48–2.44	Gln	1.89
	2.72–2.68	CA/MA/Asp	1.81	6.88–6.84	Tyr	1.77	2.68–2.64	CA/MA/Asp	1.78	2.36–2.32	Glu/MA	1.88
	2.68–2.64	CA/MA/Asp	1.80	2.32–2.28	GABA/Glu/Pro	1.76	3.00–2.96	GABA/Asn	1.74	2.28–2.24	GABA/Val	1.81
	3.00–2.96	GABA/Asn	1.77	1.36–1.32	Thr	1.70	4.72–4.68	Unannotated	1.72	1.40–1.36	Unannotated	1.81
	2.32–2.28	GABA/Glu/Pro	1.75	2.04–2.00	Glu/Pro	1.68	2.32–2.28	GABA/Glu/Pro	1.71	1.32–1.28	Thr	1.80
	1.12–1.08	Unannotated	1.74	2.28–2.24	GABA/Val	1.67	2.72–2.68	CA/MA/Asp	1.69	2.68–2.64	CA/MA/Asp	1.71
	2.28–2.24	GABA/Val	1.65	1.48–1.44	Ala	1.66	8.16–8.12	Unannotated	1.69	2.80–2.76	Asp	1.66

Table S8. NMR buckets with the highest PLS VIP values (rank 1–20) at the disease-development stage.

	Y Variable = DSIs at 3 August			*Y* Variable = DSIs at 11 August			*Y* Variable = DSIs at 17 August			*Y* Variable = DSIs at 24 August		
	**Bucket (ppm)**	**Presumed Compounds**	**VIP**	**Bucket (ppm)**	**Presumed Compounds**	**VIP**	**Bucket (ppm)**	**Presumed Compounds**	**VIP**	**Bucket (ppm)**	**Presumed Compounds**	**VIP**
Disease-development stage	3.96–3.92	Unannotated	3.05	3.96–3.92	Unannotated	2.94	3.96–3.92	Unannotated	3.02	3.96–3.92	Unannotated	2.78
	8.00–7.96	Unannotated	2.79	3.60–3.56	Suc/Fru	2.66	8.00–7.96	Unannotated	2.75	3.60–3.56	Suc/Fru	2.64
	3.60–3.56	Suc/Fru	2.58	8.00–7.96	Unannotated	2.59	3.60–3.56	Suc/Fru	2.68	8.00–7.96	Unannotated	2.58
	1.12–1.08	Unannotated	2.50	1.28–1.24	Ile	2.51	4.04–4.00	Suc/Fru	2.47	4.04–4.00	Suc/Fru	2.41
	1.28–1.24	Ile	2.49	4.04–4.00	Suc/Fru	2.46	4.00–3.96	Fru	2.46	4.00–3.96	Fru	2.33
	4.04–4.00	Suc/Fru	2.35	4.00–3.96	Fru	2.44	1.12–1.08	Unannotated	2.43	4.12–4.08	Fru	2.32
	4.00–3.96	Fru	2.34	1.12–1.08	Unannotated	2.43	1.28–1.24	Ile	2.42	1.28–1.24	Ile	2.27
	4.12–4.08	Fru	2.30	4.12–4.08	Fru	2.39	4.12–4.08	Fru	2.41	3.04–3.00	GABA	2.25
	3.04–3.00	GABA	2.14	3.04–3.00	GABA	2.23	3.04–3.00	GABA	2.20	1.20–1.16	Unannotated	2.19
	4.16–4.12	Unannotated	2.13	2.28–2.24	GABA/Val	2.04	1.20–1.16	Unannotated	2.08	2.12–2.08	Glu/Gln/Pro	2.14
	1.32–1.28	Thr	2.12	4.16–4.12	Unannotated	2.02	4.16–4.12	Unannotated	2.08	1.12–1.08	Unannotated	2.13
	2.28–2.24	GABA/Val	2.02	1.20–1.16	Unannotated	1.99	6.00–5.96	Unannotated	2.00	1.32–1.28	Thr	2.09
	5.04–5.00	Unannotated	1.99	2.12–2.08	Glu/Gln/Pro	1.95	7.16–7.12	Unannotated	1.99	2.28–2.24	GABA/Val	2.02
	1.20–1.16	Unannotated	1.97	1.32–1.28	Thr	1.91	5.04–5.00	Unannotated	1.97	4.16–4.12	Unannotated	2.01
	1.36–1.32	Thr	1.96	1.40–1.36	Unannotated	1.90	2.28–2.24	GABA/Val	1.97	5.52–5.48	Unannotated	2.00
	6.00–5.96	Unannotated	1.94	6.00–5.96	Unannotated	1.88	2.12–2.08	Glu/Gln/Pro	1.93	2.16–2.12	Glu/Gln	1.98
	7.16–7.12	Unannotated	1.89	7.16–7.12	Unannotated	1.85	1.32–1.28	Thr	1.91	2.36–2.32	Glu/MA	1.97
	2.12–2.08	Glu/Gln/Pro	1.87	1.36–1.32	Thr	1.84	1.40–1.36	Unannotated	1.87	1.36–1.32	Thr	1.95
	7.04–7.00	Unannotated	1.87	2.32–2.28	GABA/Glu/Pro	1.84	5.52–5.48	Unannotated	1.86	6.00–5.96	Unannotated	1.94
	1.40–1.36	Unannotated	1.87	8.76–8.72	Unannotated	1.84	7.04–7.00	Unannotated	1.86	2.32–2.28	GABA/Glu/Pro	1.92

Table S9. NMR buckets with the highest OPLS VIP values (rank 1–20) at the seedling stage.

	***Y* Variable = DSIs at 3 August**			***Y* Variable = DSIs at 11 August**			***Y* Variable = DSIs at 17 August**			***Y* Variable = DSIs at 24 August**		
	**Bucket (ppm)**	**Presumed Compounds**	**VIP**	**Bucket (ppm)**	**Presumed Compounds**	**VIP**	**Bucket (ppm)**	**Presumed Compounds**	**VIP**	**Bucket (ppm)**	**Presumed Compounds**	**VIP**
Seedling stage	6.24–6.20	Unannotated	2.27	6.24–6.20	Unannotated	2.25	6.24–6.20	Unannotated	2.21	5.04–5.00	Unannotated	2.46
	5.04–5.00	Unannotated	2.17	6.16–6.12	ATP	2.10	5.04–5.00	Unannotated	2.18	6.24–6.20	Unannotated	2.33
	3.96–3.92	Unannotated	2.07	5.04–5.00	Unannotated	2.06	3.96–3.92	Unannotated	2.02	3.96–3.92	Unannotated	2.27
	6.16–6.12	ATP	2.06	3.96–3.92	Unannotated	2.01	8.00–7.96	Unannotated	1.91	8.00–7.96	Unannotated	2.12
	8.00–7.96	Unannotated	1.89	8.60–8.56	ATP	1.87	6.16–6.12	ATP	1.88	0.72–0.68	Unannotated	2.06
	8.60–8.56	ATP	1.79	4.28–4.24	MA/Thr	1.78	1.32–1.28	Thr	1.83	5.08–5.04	Unannotated	2.05
	0.72–0.68	Unannotated	1.77	8.00–7.96	Unannotated	1.78	2.44–2.40	Gln	1.80	6.16–6.12	ATP	1.96
	6.76–6.72	Unannotated	1.73	0.72–0.68	Unannotated	1.77	4.28–4.24	MA/Thr	1.78	0.16–0.12	Unannotated	1.94
	9.48–9.44	Unannotated	1.73	0.16–0.12	Unannotated	1.76	0.72–0.68	Unannotated	1.77	9.28–9.24	Unannotated	1.91
	0.16–0.12	Unannotated	1.73	1.32–1.28	Thr	1.73	6.76–6.72	Unannotated	1.75	5.72–5.68	Unannotated	1.88
	0.84–0.80	Unannotated	1.71	2.44–2.40	Gln	1.72	2.64–2.600	Asp/MA	1.73	5.96–5.92	Unannotated	1.88
	1.32–1.28	Thr	1.69	5.96–5.92	Unannotated	1.70	2.00–1.96	Ile/Pro	1.73	1.32–1.28	Thr	1.80
	4.28–4.24	MA/Thr	1.66	0.84–0.80	Unannotated	1.69	1.84–1.800	Unannotated	1.69	6.76–6.72	Unannotated	1.77
	6.96–6.92	Unannotated	1.66	9.48–9.44	Unannotated	1.68	9.28–9.24	Unannotated	1.69	8.60–8.56	ATP	1.76
	9.28–9.24	Unannotated	1.64	2.64–2.60	Asp/MA	1.68	0.16–0.12	Unannotated	1.68	9.48–9.44	Unannotated	1.75
	5.96–5.92	Unannotated	1.63	5.72–5.68	Unannotated	1.67	1.36–1.32	Thr	1.68	1.56–1.52	Unannotated	1.74
	1.36–1.32	Thr	1.63	6.76–6.72	Unannotated	1.66	0.84–0.8	Unannotated	1.64	0.84–0.8	Unannotated	1.70
	2.68–2.64	CA/MA/Asp	1.62	9.28–9.24	Unannotated	1.66	9.48–9.44	Unannotated	1.61	2.00–1.96	Ile/Pro	1.69
	2.00–1.96	Ile/Pro	1.61	2.00–1.96	Ile/Pro	1.65	8.6–8.56	ATP	1.61	6.68–6.64	Unannotated	1.66
	7.04–7.00	Unannotated	1.60	1.36–1.32	Thr	1.62	6.96–6.92	Unannotated	1.61	6.96–6.92	Unannotated	1.65

Table S10. NMR buckets with the highest OPLS VIP values (rank 1–20) at the early growth stage.

	Y Variable = DSIs at 3 August			*Y* Variable = DSIs at 11 August			*Y* Variable = DSIs at 17 August			*Y* Variable = DSIs at 24 August		
	**Bucket (ppm)**	**Presumed Compounds**	**VIP**	**Bucket (ppm)**	**Presumed Compounds**	**VIP**	**Bucket (ppm)**	**Presumed Compounds**	**VIP**	**Bucket (ppm)**	**Presumed Compounds**	**VIP**
Early growth stage	3.00–2.96	GABA/Asn	2.11	3.00–2.96	GABA/Asn	2.14	3.00–2.96	GABA/Asn	2.14	3.00–2.96	GABA/Asn	2.29
	2.44–2.40	Gln	1.97	2.44–2.40	Gln	2.04	2.28–2.24	GABA/Val	2.03	2.44–2.40	Gln	2.28
	2.32–2.28	GABA/Glu/Pro	1.93	2.32–2.28	GABA/Glu/Pro	1.94	8.00–7.96	Unannotated	2.01	2.28–2.24	GABA/Val	2.11
	2.36–2.32	Glu/MA	1.92	2.36–2.32	Glu/MA	1.91	2.32–2.28	GABA/Glu/Pro	1.99	2.32–2.28	GABA/Glu/Pro	2.11
	2.28–2.24	GABA/Val	1.90	2.28–2.24	GABA/Val	1.87	5.04–5.00	Unannotated	1.97	3.76–3.72	Suc/Glc/Glu/Gln	2.01
	8.00–7.96	Unannotated	1.84	1.92–1.88	GABA/Acetate	1.86	2.44–2.40	Gln	1.97	2.48–2.44	Gln	2.00
	1.92–1.88	GABA/Acetate	1.82	8.00–7.96	Unannotated	1.79	2.36–2.32	Glu/MA	1.92	3.40–3.36	Glc	1.97
	5.04–5.00	Unannotated	1.78	3.04–3.00	GABA	1.76	1.88–1.84	GABA	1.86	2.16–2.12	Glu/Gln	1.95
	3.04–3.00	GABA	1.75	5.04–5.00	Unannotated	1.75	1.92–1.88	GABA/Acetate	1.79	3.48–3.44	Suc/Glc	1.95
	1.88–1.84	GABA	1.73	3.76–3.72	Suc/Glc/Glu/Gln	1.73	2.64–2.600	Asp/MA	1.78	5.24–5.20	Glc	1.94
	2.64–2.600	Asp/MA	1.73	2.12–2.08	Glu/Gln/Pro	1.72	6.96–6.92	Unannotated	1.76	2.40–2.36	SA/MA	1.92
	4.28–4.24	MA/Thr	1.71	2.48–2.44	Gln	1.71	2.40–2.36	SA/MA	1.75	3.44–3.40	Suc/Glc	1.91
	2.40–2.36	SA/MA	1.70	1.88–1.84	GABA	1.70	4.28–4.24	MA/Thr	1.75	2.64–2.600	Asp/MA	1.91
	3.76–3.72	Suc/Glc/Glu/Gln	1.69	2.16–2.12	Glu/Gln	1.69	2.68–2.64	CA/MA/Asp	1.73	1.88–1.84	GABA	1.91
	4.32–4.28	MA	1.69	5.72–5.68	Unannotated	1.69	7.04–7.00	Unannotated	1.72	4.28–4.24	MA/Thr	1.90
	2.48–2.44	Gln	1.68	2.64–2.60	Asp/MA	1.67	4.32–4.28	MA	1.71	4.64–4.60	Glc	1.88
	2.12–2.08	Glu/Gln/Pro	1.67	2.40–2.36	SA/MA	1.65	2.48–2.44	Gln	1.70	3.52–3.48	Glc	1.86
	2.68–2.64	CA/MA/Asp	1.67	4.28–4.24	MA/Thr	1.64	5.72–5.68	Unannotated	1.69	1.92–1.88	GABA/Acetate	1.82
	2.16–2.12	Glu/Gln	1.67	4.32–4.28	MA	1.63	1.84–1.80	Unannotated	1.68	2.12–2.08	Glu/Gln/Pro	1.82
	7.04–7.00	Unannotated	1.66	6.52–6.48	Fumarate	1.60	2.16–2.12	Glu/Gln	1.68	3.84–3.8	Suc/Glc/Fru	1.81

Table S11. NMR buckets with the highest OPLS VIP values (rank 1–20) at the root-enlargement stage.

	Y Variable = DSIs at 3 August			*Y* Variable = DSIs at 11 August			*Y* Variable = DSIs at 17 August			*Y* Variable = DSIs at 24 August		
	**Bucket (ppm)**	**Presumed Compounds**	**VIP**	**Bucket (ppm)**	**Presumed Compounds**	**VIP**	**Bucket (ppm)**	**Presumed Compounds**	**VIP**	**Bucket (ppm)**	**Presumed Compounds**	**VIP**
Root-enlargement stage	5.04–5.00	Unannotated	2.50	5.04–5.00	Unannotated	2.48	5.04–5.00	Unannotated	2.49	5.04–5.00	Unannotated	2.40
	8.00–7.96	Unannotated	2.16	2.44–2.40	Gln	2.22	8.00–7.96	Unannotated	2.18	2.44–2.40	Gln	2.25
	2.44–2.40	Gln	2.15	8.00–7.96	Unannotated	2.15	2.44–2.40	Gln	2.12	3.04–3.00	GABA	2.24
	3.04–3.00	GABA	2.10	3.04–3.00	GABA	2.14	3.04–3.00	GABA	2.09	2.32–2.28	GABA/Glu/Pro	2.19
	2.16–2.12	Glu/Gln	2.04	2.16–2.12	Glu/Gln	2.11	2.16–2.12	Glu/Gln	2.03	3.00–2.96	GABA/Asn	2.16
	2.32–2.28	GABA/Glu/Pro	2.01	2.48–2.44	Gln	2.05	2.32–2.28	GABA/Glu/Pro	2.00	2.16–2.12	Glu/Gln	2.12
	2.48–2.44	Gln	1.99	2.32–2.28	GABA/Glu/Pro	2.05	2.48–2.44	Gln	1.98	8.00–7.96	Unannotated	2.12
	3.00–2.96	GABA/Asn	1.95	3.00–2.96	GABA/Asn	2.00	6.80–6.76	Unannotated	1.95	2.48–2.44	Gln	2.07
	6.80–6.76	Unannotated	1.90	2.12–2.08	Glu/Gln/Pro	1.97	3.00–2.96	GABA/Asn	1.93	2.28–2.24	GABA/Val	2.02
	2.12–2.08	Glu/Gln/Pro	1.90	2.28–2.24	GABA/Val	1.89	3.76–3.72	Suc/Glc/Glu/Gln	1.91	3.76–3.72	Suc/Glc/Glu/Gln	2.01
	2.68–2.64	CA/MA/Asp	1.90	6.80–6.76	Unannotated	1.88	2.12–2.08	Glu/Gln/Pro	1.88	1.36–1.32	Thr	1.96
	2.28–2.24	GABA/Val	1.85	3.76–3.72	Suc/Glc/Glu/Gln	1.87	2.68–2.64	CA/MA/Asp	1.88	2.12–2.08	Glu/Gln/Pro	1.95
	3.76–3.72	Suc/Glc/Glu/Gln	1.84	2.68–2.64	CA/MA/Asp	1.85	2.28–2.24	GABA/Val	1.85	1.88–1.84	GABA	1.88
	1.48–1.44	Ala	1.80	1.48–1.44	Ala	1.82	1.48–1.44	Ala	1.81	3.48–3.44	Suc/Glc	1.86
	6.68–6.64	Unannotated	1.78	3.12–3.08	Ethanolamine	1.78	6.68–6.64	Unannotated	1.77	2.68–2.64	CA/MA/Asp	1.84
	2.72–2.68	CA/MA/Asp	1.78	2.56–2.52	CA	1.77	3.48–3.44	Suc/Glc	1.77	3.12–3.08	Ethanolamine	1.83
	2.56–2.52	CA	1.78	2.72–2.68	CA/MA/Asp	1.75	2.56–2.52	CA	1.74	4.68–4.64	Glc	1.82
	3.12–3.08	Ethanolamine	1.72	1.36–1.32	Thr	1.74	6.64–6.60	Unannotated	1.74	1.48–1.44	Ala	1.82
	6.64–6.60	Unannotated	1.71	3.48–3.44	Suc/Glc	1.72	4.68–4.64	Glc	1.74	1.32–1.28	Thr	1.81
	1.64–1.60	Unannotated	1.69	6.68–6.64	Unannotated	1.71	2.72–2.68	CA/MA/Asp	1.72	3.40–3.36	Glc	1.81

Table S12. NMR buckets with the highest OPLS VIP values (rank 1–20) at the disease-development stage.

	***Y* Variable = DSIs at 3 August**			***Y* Variable = DSIs at 11 August**			***Y* Variable = DSIs at 17 August**			***Y* Variable = DSIs at 24 August**		
	**Bucket (ppm)**	**Presumed Compounds**	**VIP**	**Bucket (ppm)**	**Presumed Compounds**	**VIP**	**Bucket (ppm)**	**Presumed Compounds**	**VIP**	**Bucket (ppm)**	**Presumed Compounds**	**VIP**
Disease-development stage	3.96–3.92	Unannotated	2.62	3.96–3.92	Unannotated	2.53	3.96–3.92	Unannotated	2.59	3.96–3.92	Unannotated	2.36
	3.60–3.56	Suc/Fru	2.38	3.60–3.56	Suc/Fru	2.46	3.60–3.56	Suc/Fru	2.45	3.60–3.56	Suc/Fru	2.34
	8.00–7.96	Unannotated	2.34	4.00–3.96	Fru	2.37	4.00–3.96	Fru	2.40	4.00–3.96	Fru	2.27
	4.00–3.96	Fru	2.31	4.04–4.00	Suc/Fru	2.33	4.12–4.08	Fru	2.35	4.12–4.08	Fru	2.24
	4.12–4.08	Fru	2.26	4.12–4.08	Fru	2.32	4.04–4.00	Suc/Fru	2.34	4.04–4.00	Suc/Fru	2.22
	1.12–1.08	Unannotated	2.26	8.00–7.96	Unannotated	2.22	8.00–7.96	Unannotated	2.32	8.00–7.96	Unannotated	2.14
	4.04–4.00	Suc/Fru	2.24	1.12–1.08	Unannotated	2.19	1.12–1.08	Unannotated	2.21	1.12–1.08	Unannotated	2.02
	4.16–4.12	Unannotated	2.12	1.28–1.24	Ile	2.11	4.16–4.12	Unannotated	2.09	3.04–3.00.00	GABA	1.99
	1.28–1.24	Ile	2.08	3.80–3.76	Suc/Glc/Fru	2.05	1.28–1.24	Ile	2.06	4.16–4.12	Unannotated	1.98
	3.80–3.76	Suc/Glc/Fru	1.99	4.16–4.12	Unannotated	2.04	3.80–3.76	Suc/Glc/Fru	2.04	2.12–2.08	Glu/Gln/Pro	1.97
	1.40–1.36	Unannotated	1.93	3.56–3.52	Suc/Glc/Fru	1.94	1.40–1.36	Unannotated	1.92	1.28–1.24	Ile	1.94
	2.12–2.08	Glu/Gln/Pro	1.88	1.40–1.36	Unannotated	1.91	3.56–3.52	Suc/Glc/Fru	1.90	2.16–2.12	Glu/Gln	1.91
	3.56–3.52	Suc/Glc/Fru	1.86	2.12–2.08	Glu/Gln/Pro	1.90	3.04–3.00	GABA	1.87	3.80–3.76	Suc/Glc/Fru	1.90
	2.16–2.12	Glu/Gln	1.83	3.04–3.00	GABA	1.88	2.12–2.08	Glu/Gln/Pro	1.87	1.40–1.36	Unannotated	1.88
	3.04–3.00	GABA	1.83	2.16–2.12	Glu/Gln	1.86	2.16–2.12	Glu/Gln	1.83	2.28–2.24	GABA/Val	1.79
	1.36–1.32	Thr	1.76	3.72–3.68	Glc/Fru	1.75	5.04–5.00		1.75	2.32–2.28	GABA/Glu/Pro	1.79
	1.32–1.28	Thr	1.76	2.44–2.40	Gln	1.69	3.72–3.68	Glc/Fru	1.74	3.56–3.52	Suc/Glc/Fru	1.79
	5.04–5.00	Unannotated	1.75	2.48–2.44	Gln	1.69	1.36–1.32	Thr	1.69	2.44–2.40	Gln	1.78
	3.72–3.68	Glc/Fru	1.73	1.36–1.32	Thr	1.68	7.04–7.00	Unannotated	1.67	1.48–1.44	Ala	1.75
	7.04–7.00	Unannotated	1.70	2.28–2.24	GABA/Val	1.66	2.28–2.24	GABA/Val	1.66	1.36–1.32	Thr	1.75

Table S13. Pearson's correlation coefficient between the original intensity of NMR buckets (normalized to the intensity of DSS) and DSIs. The buckets with |*r*| > 0.5 are listed.

**Growth Stage**	***Y* Variable = DSIs on 4 August**			***Y* Variable = DSIs on 11 August**			***Y* Variable = DSIs on 17 August**			***Y* Variable = DSIs on 24 August**		
	**Bucket (ppm)**	**Presumed Compounds**	***r***	**Bucket (ppm)**	**Presumed Compounds**	***r***	**Bucket (ppm)**	**Presumed Compounds**	***r***	**Bucket (ppm)**	**Presumed Compounds**	***r***
Seedling stage	6.24–6.20	Unannotated	0.55	9.28–9.24	Unannotated	0.53	9.28–9.24	Unannotated	0.54	5.04–5.00	Unannotated	0.54
	5.04–5.00	Unannotated	0.55	6.24–6.20	Unannotated	0.52	5.04–5.00	Unannotated	0.53	3.96–3.92	Unannotated	0.52
	3.96–3.92	Unannotated	0.55	3.96–3.92	Unannotated	0.52	3.96–3.92	Unannotated	0.52	5.96–5.92	Unannotated	−0.51
	9.28–9.24	Unannotated	0.53		Unannotated		6.76–6.72	Unannotated	0.52	0.16–0.12	Unannotated	−0.52
	6.76–6.72	Unannotated	0.51	6.16–6.12	ATP	−0.52	6.24–6.20	Unannotated	0.52			
	8.00–7.96	Unannotated	0.51									
	6.16–6.12	ATP	−0.53									
Early growth stage	8.00–7.96	Unannotated	0.59	5.04–5.00	Unannotated	0.55	5.04–5.00	Unannotated	0.57	7.56–7.52	Trp	0.54
	5.04–5.00	Unannotated	0.58	8.00–7.96	Unannotated	0.55	8.00–7.96	Unannotated	0.56	5.04–5.00	Unannotated	0.54
	0.68–0.64	DSS	0.56	0.68–0.64	DSS	0.53	0.68–0.64	DSS	0.55	8.00–7.96	Unannotated	0.52
	6.96–6.92	Unannotated	0.53	7.56–7.52	Trp	0.50	1.84–1.80	Unannotated	0.52	2.12–2.08	Glu, Gln, Pro	−0.51
	7.56–7.52	Trp	0.53	1.84–1.80	Unannotated	0.50	2.64–2.60	Asp, MA	−0.52	1.92–1.88	GABA, Acetate	−0.54
	1.84–1.80	Unannotated	0.52	2.12–2.08	Glu, Gln, Pro	−0.52	2.12–2.08	Glu, Gln, Pro	−0.52	2.28–2.24	GABA, Val	−0.55
	6.36–6.32	Unannotated	0.50	2.28–2.24	GABA, Val	−0.55	3.04–3.00	GABA	−0.53	2.32–2.28	GABA, Glu, Pro	−0.57
	3.04–3.00	GABA	−0.50	1.92–1.88	GABA, Acetate	−0.57	1.92–1.88	GABA, Acetate	−0.55	2.36–2.32	Glu, MA	−0.60
	2.12–2.08	Glu, Gln, Pro	−0.51	2.32–2.28	GABA, Glu, Pro	−0.57	5.72–5.68	Unannotated	−0.56	5.72–5.68	Unannotated	−0.63
	1.88–1.84	GABA	−0.51	5.72–5.68	Unannotated	−0.58	2.28–2.24	GABA, Val	−0.58	2.44–2.40	Gln	−0.65
	4.32–4.28	MA	−0.51	2.36–2.32	Glu, MA	−0.63	2.32–2.28	GABA, Glu, Pro	−0.59	3.00–2.96	GABA, Asn	−0.69
	2.64–2.60	Asp, MA	−0.52	2.44–2.40	Gln	−0.63	2.36–2.32	Glu, MA	−0.60			

Table S13. *Cont.*

Growth Stage	*Y* Variable = DSIs on 4 August			*Y* Variable = DSIs on 11 August			*Y* Variable = DSIs on 17 August			*Y* Variable = DSIs on 24 August		
	**Bucket (ppm)**	**Presumed Compounds**	***r***	**Bucket (ppm)**	**Presumed Compounds**	***r***	**Bucket (ppm)**	**Presumed Compounds**	***r***	**Bucket (ppm)**	**Presumed Compounds**	***r***
Early growth stage	2.40–2.36	SA, MA	−0.54	3.00–2.96	GABA, Asn	−0.69	2.44–2.40	Gln	−0.61			
	4.28–4.24	MA, Thr	−0.54				3.00–2.96	GABA, Asn	−0.66			
	5.72–5.68	Unannotated	−0.57									
	1.92–1.88	GABA, Acetate	−0.58									
	2.28–2.24	GABA, Val	−0.59									
	2.32–2.28	GABA, Glu, Pro	−0.59									
	2.44–2.40	Gln	−0.63									
	2.36–2.32	Glu, MA	−0.65									
	3.00–2.96	GABA, Asn	−0.70									
Root-enlargement stage	5.04–5.00	Unannotated	0.66	5.04–5.00	Unannotated	0.63	5.04–5.00	Unannotated	0.65	5.04–5.00	Unannotated	0.59
	8.00–7.96	Unannotated	0.57	8.00–7.96	Unannotated	0.53	8.00–7.96	Unannotated	0.56	8.00–7.96	Unannotated	0.52
	5.72–5.68	Unannotated	−0.52	2.44–2.40	Gln	−0.53	6.80–6.76	Unannotated	0.54	3.12–3.08	Ethanolamine	−0.50
	2.44–2.40	Gln	−0.52	5.72–5.68	Unannotated	−0.55	5.72–5.68	Unannotated	−0.52	2.44–2.40	Gln	−0.52
							2.44–2.40	Gln	−0.53	5.72–5.68	Unannotated	−0.59
Disease development stage	3.96–3.92	Unannotated	0.64	3.96–3.92	Unannotated	0.62	3.96–3.92	Unannotated	0.64	3.96–3.92	Unannotated	0.58
	8.00–7.96	Unannotated	0.59	3.6–3.56	Suc, Fru	0.56	8.00–7.96	Unannotated	0.58	3.60–3.56	Suc, Fru	0.56
	3.60–3.56	Suc, Fru	0.54	8.00–7.96	Unannotated	0.55	3.60–3.56	Suc, Fru	0.56	8.00–7.96	Unannotated	0.54
	1.12–1.08	Unannotated	0.52	1.28–1.24	Ile	0.53	4.04–4.00	Suc, Fru	0.52	4.04–4.00	Suc, Fru	0.51
	1.28–1.24	Ile	0.52	4.04–4.00	Suc, Fru	0.52	4.00–3.96	Fru	0.52			
				4.00–3.96	Fru	0.52	1.12–1.08	Unannotated	0.51			
				1.12–1.08	Unannotated	0.52	1.28–1.24	Ile	0.51			
				4.12–4.08	Fru	0.51	4.12–4.08	Fru	0.51			

Table S14. The NMR buckets strongly contributing to discriminating CLS resistance in sugar beet leaves.

**Growth Stage**	**Bucket (ppm)**	**Presumed Compounds**	**OPLS-VIP *Y* Valiable = DSIs on August**				**Pearson's Correlation for DSIs on August**				**Welch's *t*-Test *p*-Value**
			**3**	**11**	**17**	**24**	**3**	**11**	**17**	**24**	
Seedling stage	3.96–3.92	Unidentified	2.07	2.01	2.02	2.27	0.55	0.52	0.52	0.52	0.00404
Early growth stage	3.00–2.96	GABA, Asn	2.52	2.55	2.44	2.60	−0.70	−0.69	−0.66	−0.69	0.00011
	2.44–2.40	Gln	1.97	2.04	1.97	2.28	−0.63	−0.63	−0.61	−0.65	0.00266
	2.32–2.28	GABA, Glu, Pro	1.93	1.94	1.99	2.11	−0.59	−0.57	−0.59	−0.57	0.00048
	2.28–2.24	GABA, Val	1.90	1.87	2.03	2.11	−0.59	−0.55	−0.58	−0.55	0.00016
	1.92–1.88	GABA, Acetate	1.82	1.86	1.79	1.82	−0.58	−0.57	−0.55	−0.54	0.00356
Root-enlargement stage	5.04–5.00	Unidentified	2.50	2.48	2.49	2.40	0.66	0.63	0.65	0.59	0.00003
	8.00–7.96	Unidentified	2.16	2.15	2.18	2.12	0.57	0.53	0.56	0.52	0.00075
	2.44–2.40	Gln	2.15	2.22	2.12	2.25	−0.52	−0.53	−0.53	−0.52	0.01508
Disease-development stage	3.96–3.92	Unidentified	2.62	2.53	2.59	2.36	0.52	0.53	0.64	0.56	0.00014
	3.60–3.56	Suc/Fru	2.38	2.46	2.45	2.34	0.54	0.53	0.56	0.56	0.00314
	8.00–7.96	Unidentified	2.34	2.22	2.32	2.14	0.59	0.51	0.58	0.54	0.00199
